# Health effects of children’s summer holiday programs: a systematic review and meta-analysis

**DOI:** 10.1186/s12966-024-01658-8

**Published:** 2024-10-18

**Authors:** Emily Eglitis, Ben Singh, Timothy Olds, Rosa Virgara, Amanda Machell, Mandy Richardson, Kylie Brannelly, Aniella Grant, Jessica Gray, Terri Wilkinson, Zoe Rix, Grant R. Tomkinson, Carol Maher

**Affiliations:** 1 Alliance for Research in Exercise, Nutrition and Activity (ARENA), Allied Health and Human Performance, University of South Australia, North Terrace, Adelaide, South Australia 5000 Australia; 2 Centre for Adolescent Health, Murdoch Children’s Research Institute, Parkville, VIC Australia; 3 Department for Education, Government of South Australia, Adelaide, South Australia Australia; 4 Present address: College of Medicine and Public Health, Flinders University Sturt Road, Bedford Park, SA 5042 Australia; 5 National Outside School Hours Services Alliance, Woodend, Australia

**Keywords:** Holidays, Obesity, Physical activity, Child health, Health equity

## Abstract

**Background:**

Unfavourable changes occur in children’s health behaviours and outcomes during the summer holidays. This systematic review aimed to determine the effectiveness of summer holiday programs in mitigating these changes.

**Methods:**

Six databases (MEDLINE, JBI, PsychINFO, Embase, ERIC and Scopus) were systematically searched for experimental controlled studies that investigated programs of at least 5 days’ duration conducted exclusively during the summer holiday period on school-aged children (5–18 years). Primary outcomes were moderate-vigorous physical activity and energy intake. Secondary outcomes were sedentary behavior, diet quality, adiposity, and cardiorespiratory fitness. Risk of Bias was assessed using the PEDro tool. Effect sizes were calculated using random-effects meta-analysis with narrative synthesis of effects by student or program characteristics.

**Results:**

Ten studies (two randomised controlled trials, and eight non-randomised controlled trials) involving 1,446 participants were included. Summer programs had a significant moderate effect on reducing sedentary behaviour (*g*= -0.59, 95%CI= -1.16, -0.03) and significant small effects on improving moderate-to-vigorous physical activity (*g* = 0.35, 95%CI = 0.02, 0.67) and adiposity (*g*= -0.25, 95% CI = -0.39, -0.10). No significant change was detected for cardiorespiratory fitness (g = 0.43, 95%CI= -0.32, 1.17), energy intake (g= -0.06, 95% CI -2.33, 2.22), or diet quality (g = 0.20, 95%CI= -0.43, 0.83). Summer program effectiveness did not appear to differ by child sociodemographic or program characteristics. Concerns regarding bias and high heterogeneity impacted results.

**Conclusions:**

Summer programs show potential in promoting healthier movement behaviours in children and supporting healthy body weight during the summer months. Although evidence from the included studies has limitations, these programs produced small to moderate effect sizes and present promising health intervention opportunities for children. Future research with more rigorous study designs and comprehensive reporting is needed to confirm these findings and better understand the impact of summer programs on children’s health.

**Prospero registration:**

CRD42023409795.

**Supplementary Information:**

The online version contains supplementary material available at 10.1186/s12966-024-01658-8.

## Background

The school environment fosters a range of healthy behaviours in children, an often-overlooked benefit during the school year [1]. Curriculum components and the school environment (e.g., physical education lessons, outdoor play) along with school policies on nutrition (e.g., school lunches, canteen choices), provide opportunities to encourage physical activity (PA) and balanced diets. The structure of the school day also supports good health through regulated mealtimes and opportunities for incidental exercise (e.g., play at recess and lunch, active transport). Away from school, such as during the school holidays and on weekends, children tend to accumulate more sedentary activities and screen time, less PA, experience disrupted sleep patterns, and make poorer dietary choices compared to school days [2–6]. Summer is the longest period of the year children spend outside of school and research increasingly shows greater rates of weight gain over summer compared to the school year [7–9]. Summer holidays are contributing to the problem of childhood overweight and obesity which are major public health concerns, with comorbidities (e.g., hypertension, type 2 diabetes mellitus, cardiovascular disease, sleep apnoea) that can persist into adulthood [10, 11].

The detrimental impact of summer to children’s health is often magnified for disadvantaged children [1, 12–15]. While middle and high income families may have the resources and social structures that can mitigate some of the behaviour changes that lead to summer weight gain, poorer families often lack access to the benefits provided by the structured school environment, such as affordable childcare, nutritious meals and enriching activities (e.g., cognitively stimulating lessons, peer-group interactions, sports, arts and excursions) that are offered in summer [16]. This results in different summer experiences across different socio-economic status (SES) groups. The resulting differences in time-use over summer could impact health outcomes for disadvantaged children. If worsening health outcomes are left unaddressed and continue to accumulate annually, summer holidays could contribute to an increasing divide between the health status of high- and low-SES families [1].

Summer programs offer a promising solution to mitigate the health decline commonly observed over summer [13, 17–20]. These programs, with historical roots in the late 19th century in both the United States and European nations such as Switzerland and Germany, have gained widespread popularity [21, 22]. Transitioning from traditional residential camps to the inclusion of summer day camps, these programs have diversified, offering a range of formats and durations, facilitated by various organizations including private, religious, and nonprofit entities. The evolution and variety of summer programs highlight their potential effectiveness in combating the adverse health impacts of the summer break, providing accessible and flexible options for children across different demographics. Summer day camps employ multi-faceted approaches to improve children’s health behaviours including manipulating the physical environment (for example, providing appealing water fountains and reusable drink bottles to enhance water intake and access to gardens to increase physical activity), seeking family engagement and employing social campaigns as well directly targeting children’s behaviour through education, goal-setting and motivational strategies [23].

Despite growing research interest in the role of summer camps in preventing obesity and optimising children’s health, to date, there have been no systematic reviews evaluating the effectiveness of summer holiday programs (i.e., interventions) on modifying children’s health behaviours and important health outcomes like adiposity and cardiorespiratory fitness. In this review, we sought to address this gap, and answer the following research questions:

1. What effect do summer holiday programs have on the health behaviours and physical health of children and adolescents?
2. Do these effects differ by participant characteristics (e.g., socioeconomic disadvantage and age)?
3. Do these effects differ by program characteristics (e.g., program format, content, and duration)?

## Methods

### Protocol and registration

A systematic review protocol was informed by the Cochrane Handbook for Systematic Reviews [24] and prospectively registered with PROSPERO (Registration number: CRD42023409795) [25]. Reporting follows the PRISMA 2020 guidelines [26].

### Eligibility criteria

The PICO framework shaped our inclusion criteria. We included summer programs targeting school-aged children (5–18 years) that aimed to influence health behaviours and related outcomes, such as physical activity, diet, and weight. Programs targeting weight loss (or weight gain prevention) were included because weight loss may be a result of sustained dietary improvements. Additionally, research shows that children tend to gain weight more rapidly during the summer break, making it plausible that summer programs might aim to prevent this weight gain. Both residential and daytime programs were included if they lasted at least 5 days. While this is a short duration, it was considered adequate to demonstrate a change in the primary outcomes (diet and PA). We included experimental studies with controls not in a summer program. Primary outcomes were MVPA and diet (energy intake). Secondary outcomes were sedentary behaviour, diet quality, cardiorespiratory fitness (CRF), and adiposity. Outcomes were included if they were measured using validated, objective tools. For adiposity, both direct measures (body fat percentage) and indirect measures (BMI, BMIz) were included. Only studies published since 2000 were considered, with no language restrictions. Exclusions were made for studies where controls received a program, or the intervention wasn’t summer-exclusive. We focused on non-clinical settings, excluding medical facility studies. Full criteria are in Supplementary File 1.

### Information sources and search strategy

In April 2023, six databases were searched for peer-reviewed original articles: Embase, MEDLINE, JBI, PsychINFO (via OVID), ERIC, and Scopus, using a broad strategy developed with an academic librarian focused on population and context terms, available on SearchRxiv [27] and detailed in Supplementary File 2. Additionally, reference lists of included studies and inquiries to corresponding authors were conducted to identify further relevant studies [24].

### Selection process

Search results were imported into Endnote 20 (Clarivate Analytics, Philadelphia, PA) for duplicate removal, then into ASReview (version 1.1.1, ASReview LAB developers, Utrecht, Netherlands), an AI tool for systematic review screening validated for high accuracy (identifying 100% of eligible studies compared with manual screening [28]), for title and abstract screening by independent reviewers (EE and BS). ASReview was trained on the same five relevant studies identified during preliminary searches. Screening continued until a minimum of 10% of the total studies were screened and 100 consecutive, irrelevant titles were encountered, at which point the screening was stopped. This approach aligns with best practices suggested by ASReview to ensure a comprehensive and efficient review process [29]. The records were then processed in Covidence (Veritas Health Innovation, Melbourne, Australia) for full-text review by two independent reviewers (EE, TW, and ZR). Additionally, EE used Citationchaser (version 0.0.3, Haddaway, Grainger and Gray 2021) to search the reference lists of included studies for further relevant studies.

### Data collection process and data items

Data extraction was completed in duplicate by two independent reviewers (the primary author EE and one of TW, ZW, JG or AG) using charting tables loaded into Covidence that were piloted prior to use (Supplementary file 3). Data extraction fields included study characteristics (design, country, study aim, sample size), demographics (age, sex, SES), program details (aim, setting, features), results (outcome, measurement tool, time-points of measures, measures of effect, certainty, and statistical significance) and implementation outcomes (adverse events, attendance). Discrepancies for data extraction items and quality appraisal were solved via consensus.

### Study risk of bias assessment

The study quality was independently assessed by the primary author (EE) and one co-author (TW, ZW, JG, AG) using the valid and reliable PEDro Risk of Bias Tool [30, 31]. The PEDro tool’s maximum score is 10, indicating the lowest risk of bias. However, due to the unfeasibility of blinding in summer program studies, the scoring was adapted to a maximum of 8, excluding blinding-related items and interpreted based on previous works [32]: 7–8/8 low risk, 5–6/8 moderate risk, and 0–4/8 high risk of bias.

### Effect measures and synthesis methods

Meta-analyses were conducted for sufficiently homogeneous data from at least two studies for each of the primary and secondary outcomes. For studies with multiple intervention arms, the control group participant count was halved for each comparison to prevent over-sampling [24]. Different measurement units across studies were standardised to ensure consistency in our analysis. For instance, to convert daily percentage time in MVPA to minutes per day, we calculated the percentage of total wear time and expressed this as minutes. For accelerometers worn 24 h/day, this equates to a percentage 1440 min (24 h x 60 min = 1440 min) [33]). Similarly, where CRF was expressed as the number of completed laps on the progressive aerobic cardiovascular endurance run (PACER) [34], VO_2_max in mL/kg/min was calculated using the procedure described by Tomkinson and colleagues [35]. Where studies included insufficient details to enable meta-analysis, study authors were contacted to request further information. Effects for energy intake, sedentary behaviour and adiposity were reverse coded so that positive results for all outcomes represented an improvement.

Meta-analyses were completed using R software (version 4.3.1, using the *meta*, *metafor* and *dmetar* packages [36–40]) employing a random-effects model to account for expected heterogeneity. Standardized mean differences (SMD, Hedges’ g) were calculated, with heterogeneity assessed using a restricted maximum-likelihood estimator and Knapp-Hartung adjustments for confidence intervals (Harrer et al. 2021 [41]). Effect sizes were categorised as small (0.2), moderate (0.5) or large (0.8) [41, 42] and the findings were illustrated using forest plots. Statistical heterogeneity was evaluated using the *I*^2^ statistic. Substantial heterogeneity (*I*^2^ > 50% [24, 39]) prompted leave-one-out analyses to examine its effect on the results. Sensitivity analyses, including reanalysis excluding high bias risk studies, assessed result robustness. Due to the limited number of studies, planned publication bias analyses were not performed [24, 43].

During meta-analysis preparation for adiposity outcomes, we identified implausibly small standard deviations in Park et al. 2015 [44]. Efforts to clarify with the authors whether these figures were actually standard errors remained unanswered, so we examined heterogeneity and data patterns and compared results to adjusted analyses which considered the reported standard deviations as standard errors. This significantly improved the coherence of the meta-analytical outcomes, indicating that the reported values were likely mislabelled standard errors. Therefore, Park et al.’s data were excluded from the meta-analysis.

Results were interpreted considering the direction, magnitude and precision of the effect and overall patterns across the studies, with a statistical significance threshold of *p* = 0.05. While the PEDro tool was used to assess the rigour with which each individual study was conducted, the strength of the finding for each outcome was summarized using the Oxford Centre for Evidence Based Medicine’s (OCEBM) Levels of Evidence. Each study was assigned a Level of Evidence (1 = highest, 5 = lowest) before grading the outcome evidence A-D, factoring in the evidence level, effect consistency, and heterogeneity (with a minus sign indicating concerning levels of heterogeneity; designated as ≥ 50%) [45]. Grades were defined as A for consistent level 1 studies (e.g., single or multiple randomised controlled trials), B for level 2 (e.g., randomised trial or observational study with dramatic effects) or level 3 (e.g., non-randomised controlled cohort study) and C for level 4 (e.g., case-studies or historically controlled studies). Grade D was used for inconsistent findings across any level [46].

### Deviation from registered protocol

Subgroup analyses based on age, disadvantage, and program characteristics were not feasible due to the small number of included studies.

## Results

### Study selection

**Fig. 1:**
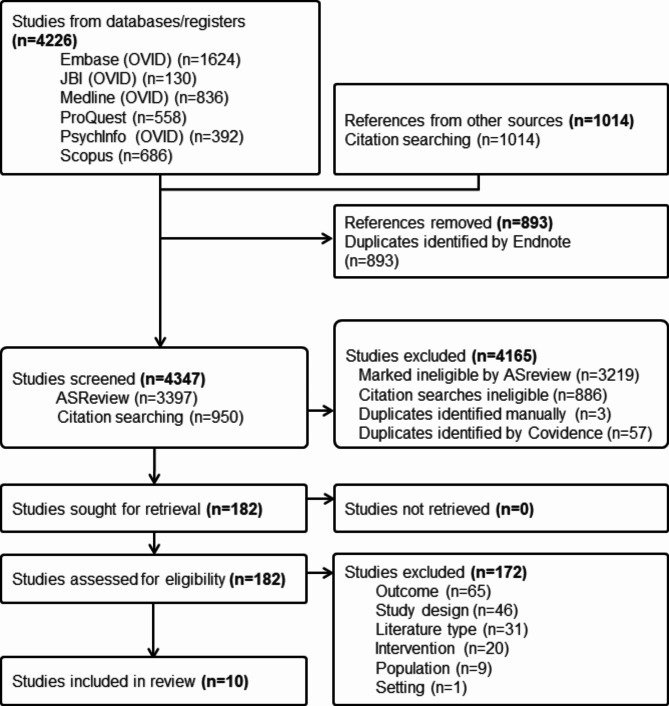
PRISMA flow diagram of study identification, screening, and inclusion

Database searching yielded 4,226 studies, with reference list searching adding 1014 more studies. After removal of duplicates, 4,347 titles and abstracts were screened, yielding 182 full texts, from which 10 studies involving 1,446 participants were included (Fig. 1). Reasons for study exclusion at full text stage are presented in Supplementary File 5.

### Study characteristics

Two studies were (individually) randomized controlled trials (RCTs) [33, 47] and 8 studies were non-randomised controlled trials [34, 44, 48–53]. Most studies were conducted in the U.S [33, 44, 47, 48, 51] (*n* = 6) with single studies from Belgium [54], Japan [50], Turkey [52] and the United Kingdom [49]. Disadvantaged populations were targeted in 50% (*n* = 5) of the studies, all from the U.S [33, 34, 44, 48, 51]. The predominant setting for the summer holiday programs was schools (*n* = 6) [34, 44, 49–52] followed by various community settings such as parks, outdoor spaces or community-based organizations (*n* = 3); [33, 48, 53] one study did not specify the setting [47]. Included studies are described in Table 1.

**Table 1:** Characteristics of included studies

First author (year) country	Study design	Participants Sample size *n*= (%female) Mean age years (SD)	Intervention: Goal, description and dose	Construct and outcome measure	Time-points measured
D’Haese (2015) Belgium [53]	Non-RCT	6–12 year-old primary school children living close to a “Play Street” *n* = 167 (45%F) Mean age: 9 (2.1)	*Goal*: Increase moderate-to vigorous-intensity PA and decreased SB *Description*: “Play Streets” organised by volunteers, supervised by parents; provided with a box of play equipment and safe play space. *Dose*: Day program, (5 h/day): 7–14 day duration (7 sessions total)	*PA*: (MVPA min/day) *SB*: (min/day) Actigraph accelerometer (hip-worn)	Baseline: “half a week” during a normal week prior to intervention. Mid-intervention: “half a week” during the play-street
Dugger (2020) USA [51]	Non-RCT	2nd -4th grade low-SES primary school students *n* = 180 (60%F) Mean age: 7.9	*Goal*: Reduce summer decline in reading achievement and mitigate accelerated unhealthy BMI gain *Description*: Healthy School Learners program led by certified teachers with periods of PA, reading, and nutrition sessions; participants received meals *Dose*: Day program (7.5 h/day): 4 days/w, 6w duration (24 sessions total)	*PA*: MVPA (min/day, total steps/day) *SB*: (min/day), *Sleep* (total min, onset, offset); Fitbit charge 2TM *Diet*: (quality): healthy & unhealthy food/drinks; Parent Report Food Screener *Screen time*: (min/day, min > 8pm); parent proxy report	Continuous measures (12 weeks) for PA, sleep. Weekly measures of screen use and diet (2x per week inc. weekdays and weekend days).
Evans (2018) USA [48]	Non-RCT	6–12 year-old children from diverse low-income backgrounds *n* = 81 (%F NR) Mean age: NR	*Goal*: Prevent summertime weight-gain *Description*: Promoting Health and Activity in Summer Trial included minimum 2 h PA and lunch run by college age staff *Dose*: Day program (4 h/day): 5 days/w, 8w duration (39 sessions total)	*PA*: (MVPA, min/day), *SB*: (min/day, %time); Actigraph Accelerometer (hip worn) *Diet*: (energy intake); 24-hr diet recalls *Adiposity*: (BMIz); height and weight calculation	Baseline (end of the school year): BMIz. Mid-intervention (weeks 4 & 5): PA & diet: Post-intervention (last week of summer): BMIz
Evans (2020) USA [33]	RCT	6–12 year-old children from racially diverse, low-income communities with high overweight/obesity prevalence *n* = 96 (58.3%F) Mean age: NR	*Goal*: Prevent excess summer weight gain by increasing PA, decreasing SB, and improving diet. *Description*: Curriculum included sports, games, obstacle courses, swimming and boating, arts and crafts with breakfast and lunch *Dose*: Day program (7.5 h/day): 7-8w duration (34–39 sessions total)	*PA*: (MVPA, min/day, %time), *SB*: (%time), Actigraph Accelerometer (wrist worn) *Diet* (energy intake, dietary quality); Healthy Eating Index *Adiposity* (BMIz)	Baseline (end of the school year): BMIz, PA, diet. Mid-intervention (weeks 4 & 5): PA & diet: Post intervention (last week of summer): BMIz
Gately (2005) UK [49]	Non-RCT	9–18 year-old children with overweight or obesity *n* = 357 (%F NR) Mean age: NR	*Goal*: Provide a safe supportive environment where children could reduce body mass whilst having fun *Description*: Structured PA, focussing on fun and skills led by PE teachers; moderate dietary restriction, and 4 educational sessions *Dose*: Residential program (6 h/day) 2-6w	*Adiposity* (BMIz, %body fat); Air displacement plethysmography *Fitness (CRF);* submaximal treadmill walking protocol	All measures taken at baseline (“start of the program”) and post-intervention (“end of the program”)
Hazar (2019) Turkey [52]	Non-RCT	7–12 year-old healthy children who enrolled in summer sports schools *n* = 132 (50%F) Mean age: 8.7 (SD NR)	*Goal*: Increase exercise to improve fitness and body weight of children *Description*: 2 h aerobic exercises in the educational play format run by sports trainers *Dose*: Day program (2 h/day): 5 days/w, 8w duration (40 sessions total)	*Body size*: Height, body weight *Fitness* (muscular); standing long jump, shot put, sit-ups, 20-m sprint)	All measures taken at baseline and post-intervention (8 weeks apart).
Matsui (2019) Japan [50]	Non-RCT	4th grade students from public elementary schools *n* = 67 (54%F) Mean age: NR	*Goal*: Maintain/PA in school aged children over summer holidays *Description*: Physical education homework for the summer vacation made up of 4 exercise programs per day with 4 exercise events *Dose*: Home-based and day program (30–90 min/day): 42 days duration	*PA*: (MVPA & LPA mins/day, steps/day), Lifecorder EX Accelerometer (waist-worn)	Measures taken at baseline (five weekdays in final week of school) & mid-intervention (14 days during summer).
Meucci (2013) USA [47]	RCT	8–12 year-old adolescents not attending structured or supervised sport activities out of school *n* = 22 (45%F) Mean age: 9.9 (1.2)	*Goal*: Have adolescents learn lifetime sport and recreational activities while increasing the time spent in exercise *Description*: Intermittent PA with play-based format plus nutrition classes; healthy snacks and lunches provided *Dose*: Day program (6 h/day): 5 days/w, 4-8w duration	*Adiposity (*BMI, %body fat); Foot-to-foot bioelectrical impedance analyser *Anthropometrics* (trunk & leg peak height velocity) *Metabolic function* (resting energy expenditure, resting heart rate); Polar Wear Link and transmitter *Fitness (CRF: peak aerobic capacity)*; direct gas analysis during modified protocol treadmill test	All measures taken at baseline (4 days pre-program) and post-intervention (within 48 h of the end of the intervention)
Park (2015) USA [44]	Non-RCT	15–17 year-old under-privileged high school students *n* = 145 (%F NR) Mean age: NR	*Goal*: Increase PA and reduce dietary intake to improve body composition and physical fitness *Description*: Aerobic exercises, resistive exercises with body weight, and a variety of games; provided with breakfast and lunch *Dose*: Day program (8 h/day): 5 days/w, 5w duration (25 sessions total)	*Body size* (height, weight) *Adiposity* (% body fat); skin fold calliper *Fitness (CRF)* VO_2_ max; Queens College step test, *(MF);* push-up test *(Flexibility)*; sit-and-reach test	All measures taken at baseline (last two weeks of school year, May) and post-intervention (first two weeks of new school year, August)
von Klingraeff (2022) USA [34]	Non-RCT	2nd -4th grade low-SES primary school students between the 25th and 75th percentile on measures of academic progress *n* = 199 (%F NR) Mean age: NR	*Goal*: Prevent accelerated BMI gain and academic learning loss over the summer holidays *Description*: Healthy School Learners program led by certified teachers with periods of PA, reading, and nutrition sessions run by teachers; participants received meals *Dose*: Day program (7.5 h/day): 4 days/w, 6w duration (24 sessions total)	*Adiposity* (BMIz) *Fitness (CRF)*; PACER laps	All measures taken at baseline (last month of the school year), post-intervention (3 months from baseline) and follow-up (12 months from baseline)

KEY

BMI: Body mass index

CRF: Cardiovascular fitness

LL: lower limb

LPA: Light physical activity

MF: Muscular fitness

MVPA: Moderate-vigorous physical activity

Non-RCT: Non-randomised control trial

NR: Not reported

PA: Physical activity

PACER: Progressive Aerobic Cardiovascular Endurance Run

RCT: Randomised control trial

SB: Sedentary Behaviour

SES: Socio-economic status

UL: upper limb

VO_2_ max: Maximal oxygen uptake

w: week

### Summer program structure, funding, and attendance

There was a great degree of diversity in the summer programs’ structure. Most programs (9/10 studies) were summer day camps that ran for between 1 and 8 weeks (median 6 weeks) [33, 34, 44, 47, 48, 51, 52]. The typical summer day camp program was offered daily (4–5 days/week) in a school or community setting for the hours of a typical school or workday. Six studies targeted healthy bodyweight: two studies involved children with overweight/obesity [33, 49], while four studies aimed to mitigate summertime weight gain in a general population [34, 48, 51, 52]. Four studies focussed on increasing PA [44, 47, 50, 54]. Children’s participation was usually funded by the research study (5/10 studies [33, 34, 47, 48, 51]), although one play-based program had existing local government funding mechanisms in place [53]. Only the residential summer camp [49] required participants to self-fund their attendance and three studies did not report funding models [44, 50, 52]. Five studies reported attendance, with three reporting higher attendance [33, 34] (66%+ of planned sessions) and two studies reporting moderate levels [48, 51, 53] (children attended 51–66% of the planned sessions). No study reported on adverse events. A summary of coding and descriptions can be found in Supplementary File 6.

**Fig. 2:**
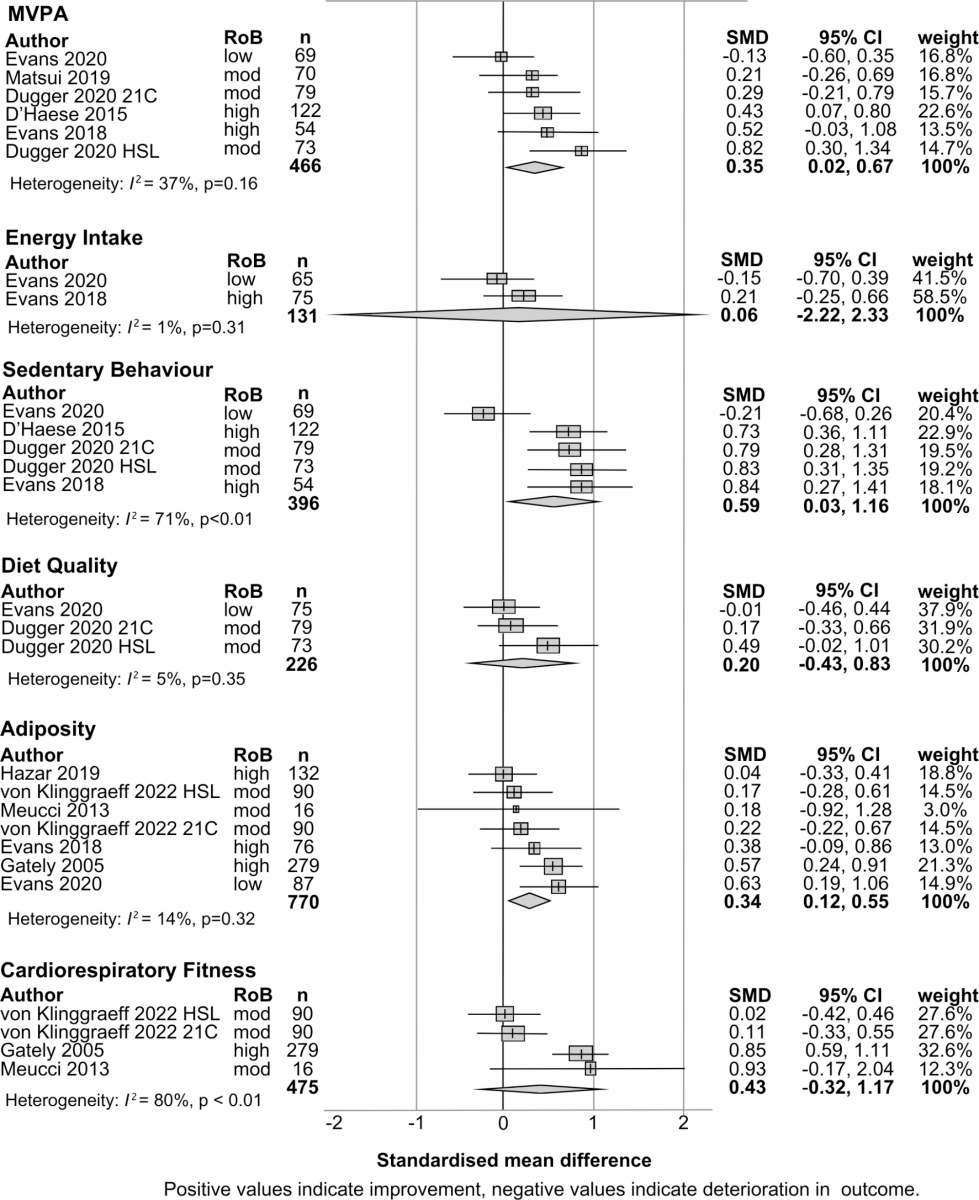
Forest plots of meta-analyses for the primary and secondary outcomes as a result of summer holiday program attendance. Results from corrected meta-analysis for CRF and adiposity presented with data from Park et al. 2015 omitted

Results from the meta-analysis of the primary and secondary outcomes are presented in Figure 2.

### Primary outcomes

Daily minutes of MVPA was meta-analysed based on five studies involving 466 participants [33, 48, 50, 51, 54]. Summer holiday programs had a significant, small-to-moderate effect on improving children’s daily MVPA levels (g = 0.35, 95%CI 0.02, 0.67, *p* = 0.04, *I*^2^ = 37%). Grade of recommendation: B (consistent level 2 and 3 studies).

Daily energy intake was meta-analysed across two studies involving 131 participants [33, 48]. Results suggested that summer holiday programs had a non-significant, negligible effect on energy intake (*g* = 0.05, 95% CI -2.22, 2.33, *p* = 0.80, *I*^2^ = 1%). Grade of recommendation: D (inconsistent results).

### Secondary outcomes

Meta-analyses were possible for four secondary outcomes: sedentary behaviour, diet quality, CRF, and adiposity.

Sedentary behaviour was meta-analysed across four studies (five comparison groups) involving 396 participants [33, 48, 51, 54]. Summer programs had a significant, moderate effect on reducing the time spent in sedentary behaviours (g = 0.59, 95%CI = 0.03, 1.16, *p* = 0.04, *I*^2^ = 71%). Grade of recommendation: Grade B- (consistent level 2 and 3 studies, with high heterogeneity).

Diet quality was meta-analysed across two studies (three comparison groups) involving 226 participants [33, 51]. Summer programs appeared to have a small, and statistically non-significant impact on diet quality (*g =* 0.20, 95%CI= -0.43, 0.83, *p* = 0.31, *I*^*2*^ = 5%). Grade of recommendation: Level B (consistent level 2 and 3 studies).

Adiposity was meta-analysed across six studies (seven comparison groups) involving 770 participants [33, 34, 47–49, 52]. Summer holiday programs had a significant, small effect on improving adiposity (*g* = 0.34, 95%CI = 0.12, 0.55, *p* = 0.01, *I*^2^ = 14%). Grade of recommendation: Level B (consistent level 2 and 3 studies).

Cardiorespiratory fitness was meta-analysed across three studies (four comparison groups) involving 475 participants [34, 47, 49]. The overall effect size suggested a small-to-moderate improvement, which was not statistically significant (*g =* 0.43, 95%CI= -0.32, 1.17, *p* = 0.17, *I*^*2*^ = 80%). Grade of recommendation: Grade B- (consistent level 2 and 3 studies, with high heterogeneity).

### Sensitivity analyses

The differential effectiveness of programs targeting children with overweight or obesity [33, 49] versus programs targeting general populations [34, 48, 52] was explored through a sensitivity analysis focussing on adiposity. The analysis revealed that interventions specifically designed for weight loss in children with overweight or obesity were more effective, producing moderate effect sizes (*g =* 0.59, 95%CI = 0.25, 0.94, *p* = 0.03, *I*^*2*^ = 0%). In contrast, interventions aimed at mitigating summer weight gain in general populations yielded small effect sizes (*g =* 0.18, 95%CI = 0.02, 0.35, *p* = 0.04, *I*^*2*^ = 0%).

### Risk of bias and heterogeneity

Risk of bias assessment revealed varying levels of bias among the included studies, reflecting differences in their methodological approach (results in Supplementary File 7). One study was deemed low risk [33], four were moderate risk [34, 47, 50, 51], and five were high risk of bias [44, 48, 49, 52, 53]. Key areas of concern related to group allocation processes; allocation concealment was achieved in only two studies [33, 51] and random allocation to intervention vs. control group was achieved in four studies [33, 34, 47, 51]. Sensitivity analyses were conducted based on risk of bias for MVPA, SB, CRF and adiposity. In all cases, the effect sizes weakened. The school holiday-related improvement in MVPA and SB were no longer statistically significant, and CRF remained not significant. For adiposity the small effect was statistically significant. Sensitivity analyses were also conducted based on heterogeneity (*I*^2^ > 50%) (also available in Supplementary file 4). Sedentary behaviour showed high statistical heterogeneity and potential causes were investigated by omitting influential studies from the analysis which increased the effect size to large (g = 0.79). Programs specifically targeting children with overweight or obesity may be focussed to a greater extent on weight loss and consequently designed and delivered in a manner that has a stronger effect on adiposity measures that may not be reflective of programs targeting the general population. Sensitivity analysis omitting these studies [33, 49] was conducted, , which decreased the effect size and it was not statistically significant (g = 0.46, 95%CI -0.18, 1.29).

### Summer holiday intervention effects and children’s socio-demographic characteristics

A narrative synthesis examined whether study effects appeared to differ based on children’s age or SES; study findings did not differ based on these factors. *Age*: Eight studies focused on primary school age children [33, 34, 47, 48, 50–53], and only these studies provided outcomes for health behaviours (i.e. results for MVPA, energy intake, SB, diet quality are all based on primary school aged children). A single study included participants from mixed age ranges [49] therefore the insufficient variation in children’s ages across the included studies did not allow for examination of differential intervention effects between older and younger children. *SES*: Regarding SES, half of the studies targeted low SES children, showing no discernible difference in MVPA, SB, or CRF effects compared to nonspecific SES groups. Dietary outcomes were solely reported in low SES-targeted studies, precluding SES-based outcome comparisons.

### Summer holiday intervention effects and program characteristics

A narrative synthesis approach was also used to examine patterns related to program characteristics such as program attendance, structure and funding. Of the five studies that reported data on attendance [33, 48, 50, 51, 54], only three reported the proportion of planned sessions that children attended overall, ranging from a mean of 50% [33] and 66% [48] to a median attendance of 75% [51]. The greatest improvements (i.e. relative reductions) in BMI were found in children who had the highest attendance levels [33, 48]. The remaining studies reported that between 80.5% and 96.4% of children accessed the program at least once over summer [50, 54]. No clear patterns were identified for program structure or funding models.

*Content*: Only one program did not provide targeted diet or PA interventions (it was a supervised play-based program [53]), but it still reported effect sizes for MVPA and SB similar to the other programs that did specifically target movement behaviours [33, 34, 44, 47–51]. While seven programs [33, 34, 44, 47–51] addressed diet behaviours, only three measured diet outcomes [33, 48, 51]. *Structure*: The single residential camp [49] produced some of the largest effect sizes for improvements in CRF and adiposity. *Frequency*: Seven of the day-programs were offered 4–5 times per week [33, 34, 44, 48, 51–53], one did not report the number of weekly sessions [47] and, despite one program being offered infrequently (i.e. once per week or less [50]), it still demonstrated a small improvement in MVPA. *Contact*: Four day-programs were delivered over a full-day [33, 34, 44, 51], one for the duration of a typical school day [47], two were half-day programs [48, 53], and two ran for 2 or less hours per visit [50, 52]. Thus, taken together, there were no clear patterns in the effects of summer holiday programs, based on the programs’ structure, funding models or children’s attendance. Behaviour changes (diet, PA, SB) were not reported for any of the studies that measured changes in CRF or adiposity.

## Discussion

Our systematic review set out to determine the impact of summer holiday programs on children’s health. The key findings were that summer programs had a small to moderate effect on increasing daily MVPA, a moderate effect on decreasing time spent in SB, and a small effect on improving children’s adiposity. Programs targeting children with overweight or obesity produced greater effects than those targeting general populations. There were no significant changes in diet (energy intake or quality) or CRF.

Results showed that summer holiday programs are effective for improving children’s PA and sedentary behaviours, but not their dietary behaviours. This is important, since increasing time spent in MVPA has beneficial effects on a variety of cardiometabolic risk factors including adiposity, glucose and lipid metabolism and resting blood pressure [55]. The effect size demonstrated here is larger than school-based PA interventions, which often yield small or inconsistent results [56]. This underscores the potential of structured programs during school breaks to maintain or enhance health benefits typically associated with school-term PA interventions.

In contrast, there was no measurable change in diet behaviours. Only two small studies reported each dietary outcome with sometimes conflicting directions of effect. This may reflect difficulties in obtaining an accurate dietary measure [57]. For example, the Parent Report Food Screener employed twice per week by Dugger and colleagues [51] may have lacked sensitivity to detect between group differences with parents less aware of children’s diet on camp days. Another explanation is that different strategies are needed than those employed during the school year. Children’s diet is relatively worse on non-school days compared to school [7] and school-based diet interventions are often effective in improving children’s diet. Similarly, summer programs also provide healthy meals and snacks, which allows control of portion sizes while also limiting unhealthy options and opportunities to snack [58, 59]. Programs with fewer daily contact hours could still allow many opportunities for children to snack at home. Potentially, summer holidays disrupt regular household routines and mean changes in the home food environment, for example, families may consume more convenient take-away meals which might off-set daytime improvements. Furthermore, only one study provided extra nutrition education [51]. Strategies that actively engage children and families may be required to influence diet behaviours over summer and more data are needed before a definitive answer can be reached on how summer programs impact children’s energy intake or diet quality.

Our review highlighted improvements in adiposity, aligning with the effects seen in school-based obesity programs [60], which can be attributed to the energy balance shift from increased PA and reduced sedentary behaviour, leading to weight loss despite stable dietary intake. Sensitivity analysis showed that programs targeting children with overweight or obesity demonstrated stronger effects than those targeting general populations. These programs focused on increasing daily PA by implementing structured activities like sports, which potentially increased vigorous PA, known to be more effective than lower intensities in reducing adiposity [61–63]. Gately et al. also introduced moderate dietary restriction but did not measure the effect on dietary outcomes while Evans et al. used the available summer food service program, similar to other interventions and did not find a change in diet quality. The exact reason for the stronger effects remains unclear as Gately et al. did not measure MVPA, and Evans et al. found a slight decrease in MVPA potentially influenced by poor attendance during the measurement week. Despite these uncertainties, the focus on PA in these programs likely played a significant role in their effectiveness.

Enhanced PA did not correspond with significant CRF improvements, which is not surprising. Various reviews on PA interventions show mixed impacts on CRF [64–69], with successful programs typically being longer, more intense, and mandatory, contrasting with our reviewed programs’ shorter span and voluntary nature. Interventions aiming to improve CRF in children generally require significantly longer durations: research indicates that achieving measurable improvements in CRF requires a minimum of seven weeks of regular, high-intensity PA [70]. Therefore, the trend here toward CRF improvement that approached significance and had a moderate effect size is worthy of brief consideration. Effective programs often featured targeted CRF components, adequate dosage, and PA-promoting strategies and settings, particularly benefiting younger, more active, or initially fitter children. The modest duration and intensity of the reviewed interventions might explain the limited CRF changes, underscoring the importance of program specifics in influencing fitness outcomes.

The effectiveness of a program could be influenced by its duration relative to the entire summer period. Dugger and colleagues [51] also compared obesogenic behaviours (PA, SB, sleep, screen time and diet) of children attending summer camp to their behaviour on non-camp days (weekdays and weekends) and found behaviours were better on camp days. Our review included programs as short as one [54], two [49] and four [47] weeks duration. In contexts where the summer holidays are long, but the intervention is short, there is ample time for children to revert to less-healthy behaviours, potentially negating some of the program’s beneficial effects. Maintaining a sustainable improvement in health-related behaviours may require consideration of the proportion of the summer holidays that the program lasts and integration of follow-up activities, supports and family engagement strategies.

This review has several strengths. It is the first to synthesize the evidence on summer holiday programs’ health behaviour and physical health impacts, during an often overlooked but substantial period of children’s lives. We focused on studies with experimental designs and applied highly rigorous systematic review methodology and synthesised the data using a meta-analysis approach. As a result, this review offers robust insights into the effectiveness of summer programs as health interventions.

There are various limitations, mostly related to the body of evidence included in this review. First, there were a relatively small number of included studies, including only two RCTs, therefore the planned sub-group meta-analysis based on participant and program characteristics had to be changed to a narrative analysis approach. Due to the small number of heterogenous studies, it wasn’t possible to determine what population or program elements may make summer holiday programs most beneficial, or to determine an accurate aggregate “exposure” variable. Attendance levels were inconsistently reported, limiting our ability to determine the level of engagement with the interventions. Consequently, the effect estimates of this review reflect an “intention to treat” approach to analysis, which typically yields smaller effects than “per protocol” analyses of participants who fully adhere to the intervention. From an implementation science perspective, the positive effects demonstrated in this review hold promise for real-life settings where variations in attendance are commonplace. We suggest that future research should prioritize detailed reporting of participant attendance and session frequency to enable more precise understanding of exposure and participants’ actual engagement. Still, there are insights available from the included studies. First, there is evidence that more frequent program attendance was related to greater health improvements [33, 48] and the single residential study produced the largest effect sizes for improvements in CRF and adiposity [49] which may reflect a larger dose or the benefit of an un-interrupted intervention. Second, exploring factors influencing attendance, it was found that attendance levels were highest amongst children whose primary carer was employed [33].

In addition, a sizable portion of studies exhibited moderate to high risk of bias, particularly affecting the analyses related to SB, MVPA and adiposity. Notably, the positive effects on SB and MVPA reduced in magnitude when the high-bias studies were excluded.

This review underscores the significance of summer holiday programs in countering the health declines children face during the extended break from school, a period noted for potential deteriorations in physical health, fitness, and adiposity [6, 8, 14, 18, 71, 72]. Such programs, traditionally viewed as childcare solutions, show promise in fostering substantial health benefits, advocating for a shift towards perceiving them as valuable public health interventions. Like school, summer programs may provide enough structure to support positive health behaviours, but summer programs also provide a break from the demands and stresses of the regimented school term. Summer programs provide fun, safe environments that enhance growth and development through play and social interaction. This reconceptualization is crucial for all stakeholders, including researchers and policymakers, aiming to mitigate the summertime regression in children’s health, especially in at-risk groups. The challenge of ensuring equitable access to these programs is highlighted, with cost and other barriers like transportation and program appeal needing to be addressed to make meaningful health interventions accessible to low-income families [73].

Future research should delineate the effective components and required intensity (“dosage”) of summer programs to optimize health benefits, necessitating more large-scale, rigorous RCTs. Implementing these studies with an emphasis on implementation science will help clarify how various factors, including adherence, acceptability, and demographics like SES and age, influence program outcomes. Collaborating with program providers and government entities is essential to ensure that effective, sustainable programs reach and benefit disadvantaged children, who are particularly vulnerable to health declines during summer breaks.

## Conclusions

Good childhood health lays the foundation for lifelong health and the prevention of chronic diseases. Summer programs show potential in promoting healthier movement behaviours in children and supporting healthy body weight during the summer months. Although the evidence from the included studies is tentative due to their limitations and the small to moderate effects observed, these programs appear to be promising health interventions for children. They present a promising strategy to combat childhood obesity by enhancing healthy behaviours during a critical time of health decline. Future research with more rigorous study designs and comprehensive reporting of exposure variables is needed to determine the program and participant characteristics of the most effective summer holiday programs. Expanding the concept of summer programs beyond enrichment or childcare services to effective public health interventions is an important consideration for policymakers and stakeholders. This approach can address summertime declines in health and to ensure that the most at-risk children can access beneficial programs.

## Electronic supplementary material

Below is the link to the electronic supplementary material.

Supplementary Material 1: PRISMA 2020 Checklist

Supplementary Material 2: Supplementary File 1: Inclusion/Exclusion criteria

Supplementary Material 3: Supplementary File 2: Search Strategy

Supplementary Material 4: Supplementary File 3: Example data extraction form

Supplementary Material 5: Supplementary File 4: Meta Analysis Summary of Results with Sensitivity Analysis

Supplementary Material 6: Supplementary File 5: Reasons for study exclusion during full text screening

Supplementary Material 7: Supplementary File 6: Thematic Coding of Program Characteristics

Supplementary Material 8: Supplementary File 7: Quality appraisal of included studies

## Data Availability

The datasets analysed in this review are derived from publicly available sources. All data can be accessed through the original publications. The search strategy is available on Search Rxiv [27].

## Abbreviations

BMI: Body Mass Index
CVF: Cardiovascular Fitness
LL: Lower Limb
LPA: Light Physical Activity
MF: Muscular Fitness
MVPA: Moderate-to-Vigorous Physical Activity
Non-RCT: Non-Randomised Control Trial
NR: Not Reported
PA: Physical Activity
PACER: Progressive Aerobic Cardiovascular Endurance Run
RCT: Randomised Controlled Trials
SB: Sedentary Behavior
SES: Socioeconomic Status
UL: Upper Limb
US: United States
w: week/s
