# Supplementary material for: Health effects of children’s summer holiday programs: a systematic review and meta-analysis
Source: Int J Behav Nutr Phys Act. 2024 Oct 18;21:119. doi: 10.1186/s12966-024-01658-8 (PMC11488216; doi:10.1186/s12966-024-01658-8)
Supplement: Supplementary file 2 — Supplementary Material 2: Supplementary File 1: Inclusion/Exclusion criteria [file 12966_2024_1658_MOESM2_ESM.docx]

**Supplementary File 1: Inclusion/Exclusion criteria**

**Summary Table: Overview of the inclusion/exclusion criteria using the PICO framework** - Physical Health

|  | **Inclusion** | **Exclusion** |
| --- | --- | --- |
| Populations/ participants | Children in years kindergarten to grade 12 | Clinical populations (e.g., diabetes, cancer), special needs (learning or intellectual disabilities), gifted/especially talented (e.g., elite athlete training camps). |
| Intervention | Summer holiday programs: Programs with a duration of at least 5 days conducted over the summer holiday period. Programs may include day or overnight or residential programs. | Program evaluation with no health outcomes. Studies conducted in a clinical setting. |
| Comparators | Control group not receiving a summer program. | Comparison group who received another intervention. |
| Outcome(s) | *Primary outcome*:   1. Physical activity 2. Diet     *Secondary outcomes*:   1. Adiposity 2. Cardiorespiratory fitness 3. Sleep 4. Sedentary behaviour 5. Screen time     *Implementation outcomes:* Adverse events |  |
| Study designs | Experimental studies (e.g., randomized and non-randomized, controlled trials). |  |
| Publication  types | Full length, peer-reviewed original research articles published from the year 2000 inclusive. No language limits. | Grey literature, conference abstracts, dissertations. Studies published before the year 2000. Review papers. |
