# Supplementary material for: Health effects of children’s summer holiday programs: a systematic review and meta-analysis
Source: Int J Behav Nutr Phys Act. 2024 Oct 18;21:119. doi: 10.1186/s12966-024-01658-8 (PMC11488216; doi:10.1186/s12966-024-01658-8)
Supplement: Supplementary file 3 — Supplementary Material 3: Supplementary File 2: Search Strategy [file 12966_2024_1658_MOESM3_ESM.docx]

**Supplementary File 2: Search Strategy**

| Medline (OVID) | 1 | Child/ |
| --- | --- | --- |
|  | 2 | Adolescent/ |
|  | 3 | (child* or adolescen* or teen* or youth* or young people or young person or boy or girl).ti,ab,kf. |
|  | 4 | Holidays/ |
|  | 5 | ((summer or school) adj2 (holiday* or vacation*)).ti,ab,kf. |
|  | 6 | 1 OR 2 OR 3 |
|  | 7 | 4 OR 5 |
|  | 8 | 6 AND 7 |
|  | 9 | limit 9 to yr="2000 -Current" |
| PsychINFO (OVID) | 1 | Adolescent Behavior/ or Adolescent Attitudes/ or Adolescent Psychology/ or Adolescent Health/ |
|  | 2 | Child Care/ or Child Behavior/ or Child Attitudes/ or Child Psychology/ or Child Health/ |
|  | 3 | child.mp. |
|  | 4 | adolescent.mp. |
|  | 5 | (child* OR adolescen* OR boy OR girl).ti,ab,tw. |
|  | 6 | 1 OR 2 OR 3 OR 4 OR 5 |
|  | 7 | Holidays/ |
|  | 8 | ((school* or summer*) adj2 (holiday* or vacation*)).ti,ab,tw. |
|  | 9 | (summer adj2 (months or period or learning or "between grade*")).ti,ab,tw. |
|  | 10 | 7 OR 8 OR 9 |
|  | 11 | 6 AND 10 |
|  | 12 | limit 11 to yr="2000 -Current" |
| Embase | 1 | school child/ |
|  | 2 | adolescent/ |
|  | 3 | (child* or adolescen*).ti,ab,kf. |
|  | 4 | 1 or 2 or 3 |
|  | 5 | ((Summer* OR school*) adj2 (holiday* OR Vacation*)).ti,ab,kf. |
|  | 6 | (summer adj2 (months OR period OR learning OR "between grade*")).ti,ab,kf. |
|  | 7 | 5 OR 6 |
|  | 8 | 4 AND 7 |
|  | 9 | limit 8 to yr="2000 -Current" |
| JBI | 1 | (child* or adolescen* or student* or grade* or boy* or girl* or youth).mp. |
|  | 2 | (Holiday* or vacation* or summer*).mp. |
|  | 3 | 1 and 2 |
|  | 4 | limit 3 to yr="2000 -Current" |
| ERIC |  | (((mainsubject(Child) OR mainsubject(Adolescent) OR mainsubject(Youth)) OR (TI,AB,IF(Children OR child* OR adolescen* OR "boy" OR "girl") OR TI,AB,IF(student* OR Grade*) OR TI,AB,IF(youth))) AND (mainsubject(vacations) OR TI,AB,IF((school*) NEAR/2 (Vacation* OR holiday* OR Summer*)) OR TI,AB,IF((summer) NEAR/2 (Vacation* OR months OR holiday OR period OR school)))) AND (pd(20000101-20230331) AND PEER(yes)) |
| Scopus |  | ( TITLE-ABS-KEY ( child*  OR  adolescen*  OR  boys  OR  girls ) )  AND  ( TITLE-ABS-KEY ( ( summer*  W/2  ( holiday*  OR  vacation*  OR  school* ) ) ) )  AND  PUBYEAR  >  1999  AND  PUBYEAR  <  2024  AND  ( LIMIT-TO ( DOCTYPE ,  "ar" ) ) |

Search Strategy also available on SearchRxiv: <https://doi.org/10.1079/searchRxiv.2023.00380>
