# Supplementary material for: Health effects of children’s summer holiday programs: a systematic review and meta-analysis
Source: Int J Behav Nutr Phys Act. 2024 Oct 18;21:119. doi: 10.1186/s12966-024-01658-8 (PMC11488216; doi:10.1186/s12966-024-01658-8)
Supplement: Supplementary file 4 — Supplementary Material 4: Supplementary File 3: Example data extraction form [file 12966_2024_1658_MOESM4_ESM.docx]

**Supplementary File 3: Example data extraction form**

| Study title |  |
| --- | --- |
| Year of publication |  |
| Lead author email address |  |
| Country in which the study was conducted |  |
| Reviewer |  |
| Study design |  |

**Population description**

|  | sample size n= | sex female n= | female % (whole number) | range (years) | age mean (years) | Age (SD) (years) |
| --- | --- | --- | --- | --- | --- | --- |
| Whole sample |  |  |  |  |  |  |
| Intervention group |  |  |  |  |  |  |
| Alternative intervention group (if applicable) |  |  |  |  |  |  |
| Control group |  |  |  |  |  |  |

**Program Features**

| Method of recruitment of participants |  |
| --- | --- |
| Population SES |  |
| Goal of the program |  |
| Intervention Program Description |  |
| What did the comparison group receive? |  |

**Program structure – setting and delivery**

*Intervention group*

| Setting |  |
| --- | --- |
| Delivery |  |
| Cost |  |
| Format: day/residential |  |
| Duration (days/week, total weeks from start to finish) |  |
| Duration per day (hrs) |  |
| Number of sessions |  |
| Other program features |  |

**Outcome measured & tool used**

- Physical Health:
- Health Behaviour:

Physical Health Outcome

|  | Outcome | N= | baseline mean (variability metric and value) | end program mean (variability metric and value) | Effect size, test statistic and value | confidence intervals (lower and upper) | Follow-up measures? (timepoint) | Follow-up outcomes (change, effect, significance, CI) |
| --- | --- | --- | --- | --- | --- | --- | --- | --- |
| Intervention group |  |  |  |  |  |  |  |  |
| Control group |  |  |  |  |  |  |  |  |

*Notes*

Health Behaviour Outcome

|  | Outcome | N= | baseline mean (variability metric and value) | end program mean (variability metric and value) | Effect size, test statistic and value | confidence intervals (lower and upper) | Follow-up measures? (timepoint) | Follow-up outcomes (change, effect, significance, CI) |
| --- | --- | --- | --- | --- | --- | --- | --- | --- |
| Intervention group |  |  |  |  |  |  |  |  |
| Control group |  |  |  |  |  |  |  |  |

*Notes*

**Subgroup analyses by SES or race (& results)?**

**Implementation outcomes**

|  | Attendance level | Incentives to attend | Adverse events | Funding for the program | Conflicts of interests |
| --- | --- | --- | --- | --- | --- |
| Intervention group |  |  |  |  |  |
| Alternative intervention (if applicable) |  |  |  |  |  |

Any notes you would like the other authors to know?

Summarize the paper's main findings:
