# Supplementary material for: Health effects of children’s summer holiday programs: a systematic review and meta-analysis
Source: Int J Behav Nutr Phys Act. 2024 Oct 18;21:119. doi: 10.1186/s12966-024-01658-8 (PMC11488216; doi:10.1186/s12966-024-01658-8)
Supplement: Supplementary file 5 — Supplementary Material 5: Supplementary File 4: Meta Analysis Summary of Results with Sensitivity Analysis [file 12966_2024_1658_MOESM5_ESM.docx]

**Supplementary File 4: Meta Analysis Summary of Results with Sensitivity Analysis**

**Primary Outcomes**

| **Analysis** | ***g*** | **95% CI** | ***p*** | **95%PI** | **I^2^** | **95%CI** |
| --- | --- | --- | --- | --- | --- | --- |
| *MVPA* | | | | | | |
| Random effects | 0.349 | 0.024, 0.674 | 0.040 | -0.263, 0.961 | 37.3% | 0%, 75% |
| Fixed effects | 0.347 |  |  |  |  |  |
| Influential Cases Removed | 0.367 | 0.162, 0.572 | 0.011 | -0.133, 0.867 | 0.0% | 0%, 85% |
| High Risk of Bias Removed | 0.289 | -0.330, 0.908 | 0.234 | -1.218, 1.796 | 57.4% | 0%, 86% |
| *Diet – Energy Intake* | | | | | | |
| Random effects | 0.059 | -2.215, 2.334 | 0.797 | (n/a) | 1.3%; | (n/a) |
| Fixed effects | 0.0597 |  |  |  |  |  |
| Influential Cases Removed | n/a |  |  |  |  |  |
| High Risk of Bias Removed | n/a |  |  |  |  |  |

**Sensitivity Analyses**

| **Outcome** | **Influential cases** | **High Risk of Bias (RoB) studies** |
| --- | --- | --- |
| MVPA | Dugger 2020HSL, Evans 2020 | D’Haese 2015, Evans 2018 |
| Diet - Energy | n/a (n=2 studies) | n/a (n=2 studies) |

**Secondary Outcomes**

| **Analysis** | ***g*** | **95% CI** | ***p*** | **95%PI** | **I^2^** | **95%CI** |
| --- | --- | --- | --- | --- | --- | --- |
| *Sedentary Behaviour* | | | | | | |
| Random effects | 0.591 | 0.025, 1.157 | 0.044 | -0.793, 1.975 | 70.8% | 26%, 89% |
| Fixed effects | 0.586 |  |  |  |  |  |
| Influential Cases Removed | 0.785 | 0.702, 0.868 | < 0.0001 | 0.265, 1.306 | 0.0% | 0%, 85% |
| High Risk of Bias Removed | 0.464 | -1.011, 1.939 | 0.3086 | -7.662, 8.590 | 82.2% | 45%, 94% |
| *Diet - Quality* | | | | | | |
| Random effects | 0.200 | -0.432, 0.831 | 0.307 | -1.805, 2.204 | 5.2% | 0%, 90% |
| Fixed effects | 0.057 |  |  |  |  |  |
| Influential Cases Removed | n/a |  |  |  |  |  |
| High Risk of Bias Removed | n/a |  |  |  |  |  |
| *Adiposity* |  |  |  |  |  |  |
| Random effects | 0.335 | 0.117, 0.554 | 0.009 | -0.094, 0.764 | 14% | 0%, 75% |
| Fixed effects | 0.340 |  |  |  |  |  |
| High Risk of Bias Removed | 0.333 | 0.042, 0.709 | 0.067 | -0.368, 1.034 | 0% | 0%, 85% |
| *Sensitivity analysis: adiposity* |  |  |  |  |  |  |
| Interventions targeting weight loss^1^ (k=2) | 0.593 | 0.246, 0.940 | 0.029 | n/a | 0% | n/a |
| Interventions targeting general populations^2^ (k=5) | 0.182 | 0.015, 0.348 | 0.039 | -0.159, 0.522 | 0% | 0%, 79% |
| *Cardiorespiratory fitness* |  |  |  |  |  |  |
| Random effects | 0.426 | -0.316, 1.169 | 0.165 | -1.607, 2.459 | 80% | 47%, 93% |
| Fixed effects | 0.540 |  |  |  |  |  |
| Influential Cases Removed | 0.128 | -0.576, 0.832 | 0.517 | -1.812, 2.068 | 13% | 0%, 91% |
| High Risk of Bias Removed | (as above) | | | | | |

1. Studies targeting weight loss in children with OW/OB: Gately 2005, Evans 2020
2. Studies targeting PA or weight maintenance in general populations: Evans 2018, Hazar 2019, Meucci 2019, von Klinggraeff 2022

**Sensitivity Analyses**

| **Outcome** | **Influential cases** | **Studies with high risk of bias** **(RoB)** |
| --- | --- | --- |
| SB | Evans 2020 | D’Haese 2015, Evans 2018 |
| Diet – Quality | n/a (heterogeneity <50%) | n/a (No high RoB studies) |
| Adiposity | n/a (heterogeneity <50%) | Hazar 2019, Evans 2018, Gately 2005 |
| CRF | Gately 2005 | Gately 2005 |
