# Supplementary material for: Health effects of children’s summer holiday programs: a systematic review and meta-analysis
Source: Int J Behav Nutr Phys Act. 2024 Oct 18;21:119. doi: 10.1186/s12966-024-01658-8 (PMC11488216; doi:10.1186/s12966-024-01658-8)
Supplement: Supplementary file 6 — Supplementary Material 6: Supplementary File 5: Reasons for study exclusion during full text screening [file 12966_2024_1658_MOESM6_ESM.docx]

**Supplementary File 5: Reasons for study exclusion during full text screening**

| Title | Authors | Published  Year | Exclusion  Reason |
| --- | --- | --- | --- |
| Active generations: An intergenerational approach to preventing childhood obesity | Werner, D.', 'Teufel, J.', 'Holtgrave, P. L.', 'Brown, S. L.' | 2012 | Wrong study design |
| Active summers matter: evaluation of a community-based summertime program targeting obesogenic behaviors of low-income, ethnic minority girls | Bohnert, A. M.', 'Ward, A. K.', 'Burdette, K. A.', 'Silton, R. L.', 'Dugas, L. R.' | 2014 | Wrong study design |
| Addressing Childhood Hunger during the Summer Months: Using Gleaned Produce for Snacks and Interactive Nutrition Education on Food Systems and Healthy Eating | Oo, K.', 'Stephenson, T.', 'Hege, A.', 'Brewer, D.', 'Gamboa, L.', 'Hildesheim, L.', 'Serra, L.', 'Houlihan, J.', 'Koempel, A.' | 2020 | Wrong outcomes |
| Camp NERF: Caregiver outcomes from a theory-based nutrition education recreation and fitness program aimed at preventing unhealthy weight gain in underserved children during summer months | Hopkins, L. C.', 'Webster, A.', 'Sharn, A.', 'Gunther, C.' | 2017 | Wrong literature type |
| Camp NERF: methods of a theory-based nutrition education recreation and fitness program aimed at preventing unhealthy weight gain in underserved elementary children during summer months | Hopkins, L. C.', 'Fristad, M.', 'Goodway, J. D.', 'Eneli, I.', 'Holloman, C.', 'Kennel, J. A.', 'Melnyk, B.', 'Gunther, C.' | 2016 | Wrong literature type |
| Can an Immersion in Wellness Camp Influence Youth Health Behaviors? | Mabary-Olsen, Elizabeth A.', 'Litchfield, Ruth E.', 'Foster, Randal', 'Lanningham-Foster, Lorraine', 'Campbell, Christina' | 2015 | Wrong outcomes |
| Changes in Daily Step Counts and Health-Related Fitness after a Sports-Based Residential Summer Camp in Boys | Wahl-Alexander, Zachary', 'Brusseau, Timothy', 'Burns, Ryan' | 2020 | Wrong study design |
| Characteristics of effective summer learning programs in practice | Bell, S. R.', 'Carrillo, N.' | 2007 | Wrong literature type |
| Cognitive outcomes from the Game-Design and Learning (GDL) after-school program | Akcaoglu, M.', 'Koehler, M. J.' | 2014 | Wrong intervention |
| Combating Child Summer Food Insecurity: Examination of a Community-Based Mobile Meal Program | Bruce, J. S.', 'De La Cruz, M. M.', 'Lundberg, K.', 'Vesom, N.', 'Aguayo, J.', 'Merrell, S. B.' | 2019 | Wrong study design |
| Come together, play, be active: Physical activity engagement of school-age children at Play Streets in four diverse rural communities in the U.S | Umstattd Meyer, M. R.', 'Bridges Hamilton, C. N.', 'Prochnow, T.', 'McClendon, M. E.', 'Arnold, K. T.', 'Wilkins, E.', 'Benavidez, G.', 'Williams, T. D.', 'Abildso, C. G.', 'Pollack Porter, K. M.' | 2019 | Wrong study design |
| A comparison of a gardening and nutrition program with a standard nutrition program in an out-of-school setting | Poston, S. A.', 'Shoemaker, C. A.', 'Dzewaltowski, D. A.' | 2005 | Wrong intervention |
| Complete EATS: Summer meals offered by the emergency department for food insecurity | Cullen, D.', 'Blauch, A.', 'Mirth, M.', 'Fein, J.' | 2019 | Wrong setting |
| Computing goals, values, and expectations: Results from an after-school program for girls | Denner, J.', 'Werner, L.', 'Martinez, J.', 'Bean, S.' | 2012 | Wrong outcomes |
| Contribution of children's reading motivation and prosocial efficacy to reading growth | Ha, C.', 'Roehrig, A. D.' | 2022 | Wrong outcomes |
| A Creative Strengths-Based Group Counseling Intervention for African American Boys | Prasath, P. R.', 'Steen, S.', 'McVay, K.' | 2023 | Wrong outcomes |
| Culturally relevant reading for supporting underserved children's prosocial self-efficacy and reading skills: A transformative social-emotional learning approach | Ha, Cheyeon' | 2023 | Wrong literature type |
| Development and evaluation of WillTry. An instrument for measuring children's willingness to try fruits and vegetables | Thomson, J. L.', 'McCabe-Sellers, B. J.', 'Strickland, E.', 'Lovera, D.', 'Nuss, H. J.', 'Yadrick, K.', 'Duke, S.', 'Bogle, M. L.' | 2010 | Wrong study design |
| The diet of children attending a holiday programme in the uk: Adherence to uk food-based dietary guidelines and school food standards | Crilley, E.', 'Brownlee, I.', 'Defeyter, M. A.' | 2022 | Wrong intervention |
| Diet of children under the government-funded meal support program in Korea | Kwon, Sooyoun', 'Lee, Kiwon', 'Yoon, Jihyun' | 2010 | Wrong intervention |
| Digital Game Building: Learning in a Participatory Culture | Li, Qing' | 2010 | Wrong study design |
| Do you hear what I hear? Overweight children's perceptions of different physical activity settings | Meaney, K. S.', 'Hart, A. M.', 'Griffin, L. K.' | 2011 | Wrong outcomes |
| Effect of sleep intervention using a summer holiday workbook for junior high-school students | Oka, Y.', 'Horiuchi, F.', 'Kawabe, K.' | 2015 | Wrong literature type |
| The Effect of Teacher-Family Communication on Student Engagement: Evidence from a Randomized Field Experiment | Kraft, Matthew A.', 'Dougherty, Shaun M.' | 2013 | Wrong outcomes |
| Effectiveness of an intervention program for six year olds: A summer-school model | Bekman, S.', 'Aksu-KoÃ§, A.', 'Erguvanli-Taylan, E.' | 2012 | Wrong outcomes |
| Effectiveness of an intervention program for six-year-olds: A summer-school model | Bekman, S.', 'Aksu-KoÃ§, A.', 'Erguvanli-Taylan, E.' | 2011 | Wrong intervention |
| Effectiveness of the bicisalud programme in a group of schoolchildren with excess weight | PÃ©rez, E. R. S.', 'Aranzamendi, J. I. L.', 'Cano, M. R. M.', 'Delgado, D. B.', 'CÃ¡mara, G. L. C.' | 2018 | Wrong study design |
| Effects of a practice-focused nutrition intervention in Hungarian adolescents | Takacs, H.', 'Martos, E.', 'Anna Kovacs, V.' | 2020 | Wrong intervention |
| Effects of a summer school-readiness programme on measures of literacy and behaviour growth: A pilot study | McLeod, Ragan', 'Kim, Sunyoung', 'Tomek, Sara', 'McDaniel, Sara' | 2019 | Wrong population |
| Effects of pairing aggressive and nonaggressive children in strategic peer affiliation | Hektner, J. M.', 'August, G. J.', 'Realmuto, G. M.' | 2003 | Wrong outcomes |
| Effects of Recreational exercises on Childrenâ€™s Anthorpometric Parameters and their level of nutrition knowledge | Karacabey, K.', 'Derdin, M.' | 2014 | Wrong study design |
| The energykids pilot study: Comparing energy balance of primary school children during school and summer camp | Franchini, C.', 'Rosi, A.', 'Ricci, C.', 'Scazzina, F.' | 2021 | Wrong study design |
| The EnergyKids project: Pilot study on the energy balance of primary school children during school days and summer camp days | Rosi, A.', 'Franchini, C.', 'Scazzina, F.' | 2020 | Wrong literature type |
| Engaging Excellent Aboriginal Students in Science: An Innovation in Culturally-Inclusive Schooling | Aldous, Carol', 'Barnes, Alan', 'Clark, Julie' | 2008 | Wrong study design |
| Environmental and social-motivational contextual factors related to youth physical activity: Systematic observations of summer day camps | Zarrett, N.', 'Sorensen, C.', 'Skiles, B.' | 2013 | Wrong study design |
| Evaluation of a five-day recipe booklet for enhancing the use of fruits and vegetables in low-income households | Birmingham, Brenda', 'Shultz, Jill Armstrong', 'Edlefsen, Miriam' | 2004 | Wrong outcomes |
| An evaluation of a parent tutoring reading fluency program | Mitchell, Rachel Courtney' | 2013 | Wrong literature type |
| Examining the impact of a summer learning program on children's weight status and cardiorespiratory fitness: A natural experiment | Hunt, E. T.', 'Whitfield, M. L.', 'Brazendale, K.', 'Beets, M. W.', 'Weaver, R. G.' | 2019 | Wrong study design |
| Examining the potential protective effect of structured programming on child weight during the summer months through intervention and observational research: Camp NERF (nutrition, education, recreation, and fitness) and project SWEAT (summer weight and environmental assessment trial) | Hopkins, Laura C.' | 2019 | Wrong literature type |
| Experiencing nature in children's summer camps: Affective, cognitive and behavioural consequences | Collado, Silvia', 'Staats, Henk', 'Corraliza, Jose A.' | 2013 | Wrong outcomes |
| An exploratory study comparing students' science identity perceptions derived from a hands-on research and nonresearch-based summer learning experience | Hernandez-Matias, L.', 'Perez-Donato, L.', 'Roman, P. L.', 'Laureano-Torres, F.', 'Calzada-Jorge, N.', 'Mendoza, S.', 'Washington, A. V.', 'Borrero, M.' | 2020 | Wrong outcomes |
| A fact-finding study concerning the pro-environmental behavior of elementary school students and guardians: First analysis of participants in Kyoto Prefecture Energy Conservation Challenge during summer vacation | Kim, Y.', 'Kihara, H.', 'Matsubara, N.' | 2019 | Wrong study design |
| Familias Unidas: The efficacy of an intervention to promote parental investment in Hispanic immigrant families | Pantin, Hilda', 'Coatsworth, J. Douglas', 'Feaster, Daniel J.', 'Newman, Frederick L.', 'Briones, Ervin', 'Prado, Guillermo', 'Schwartz, Seth J.', 'Szapocznik, Jose' | 2003 | Wrong intervention |
| Families, Schools, and Summer Learning | Borman, Geoffrey D.', 'Benson, James', 'Overman, Laura T.' | 2005 | Wrong outcomes |
| Food Insecurity: A Constant Factor in the Lives of Low-Income Families in Scotland and England | Shinwell, Jackie', 'Defeyter, Margaret Anne' | 2021 | Wrong population |
| Food safety and sustainable nutrition workshops: Educational experiences for primary school children in Turin, Italy | Traversa, A.', 'Adriano, D.', 'Bellio, A.', 'Bianchi, D. M.', 'Gallina, S.', 'Ippolito, C.', 'Romano, A.', 'Durelli, P.', 'Pezzana, A.', 'Decastelli, L.' | 2017 | Wrong intervention |
| Fostering Healthy Development among Middle School Females: A Summer Program | Caton, Mary', 'Field, Julaine E.', 'Kolbert, Jered B.' | 2010 | Wrong literature type |
| From frontal teaching to emotional understanding - A modern concept in childhood obesity therapy | Katrin, H.', 'Dirk, B.', 'Anneco, D.', 'Bjorn, B.', 'Hanna, S.', 'Dagmar, L.' | 2017 | Wrong literature type |
| From Global to Local | Kye, Hannah' | 2019 | Wrong literature type |
| From I to We: Collaboration in Entrepreneurship Education and Learning? | Warhuus, Jan P.', 'Tanggaard, Lene', 'Robinson, Sarah', 'ErnÃ¸, Steffen Moltrup' | 2017 | Wrong population |
| The girls creating games program: An innovative approach to integrating technology into middle school | Denner, J.' | 2007 | Wrong outcomes |
| Halting the Summer Achievement Slide: A Randomized Field Trial of the KindergARTen Summer Camp | Borman, Geoffrey D.', 'Goetz, Michael E.', 'Dowling, N. Maritza' | 2009 | Wrong outcomes |
| Healthy lifestyle intervention for obese children and their families as a part of the preventive health care programme for children and adolescents | Homsak, M.', 'Truden-Dobrin, P.', 'Vogrin, B.', 'Kotnik, P.', 'Pibernik, T.' | 2021 | Wrong literature type |
| High intensity interval training vs. high-volume running training during pre-season conditioning in high-level youth football: a cross-over trial | Faude, O.', 'Schnittker, R.', 'Schulte-Zurhausen, R.', 'Muller, F.', 'Meyer, T.' | 2013 | Wrong population |
| High-intensity interval training for overweight adolescents: Program acceptance of a media supported intervention and changes in body composition | Herget, S.', 'Reichardt, S.', 'Grimm, A.', 'Petroff, D.', 'KÃ¤pplinger, J.', 'Haase, M.', 'Markert, J.', 'BlÃ¼her, S.' | 2016 | Wrong outcomes |
| Holiday Club Programmes in Northern Ireland: The Voices of Children and Young People | Shinwell, Jackie', 'Finlay, Ellen', 'Allen, Caitlin', 'Defeyter, Margaret Anne' | 2021 | Wrong study design |
| Ignite the Leader Within: Virtual Latinx Youth Empowerment and Community Leadership amid COVID-19 | Montes, Pablo', 'Bourommavong, Monica', 'Landeros, Judith', 'Urrieta, Luis, Jr.', 'Robinson, Courtney' | 2021 | Wrong outcomes |
| The Immediate and Lasting Effects of Resident Summer Camp on Movement Behaviors Among Children | Kidokoro, Tetsuhiro', 'Minatoya, Yuji', 'Imai, Natsuko', 'Shikano, Akiko', 'Noi, Shingo' | 2022 | Wrong study design Emily Eglitis (2023-09-21 12:45:04)(Included): Not an RCT or non-randomised CT |
| Impact of a year-round school calendar on children's BMI and fitness: Final outcomes from a natural experiment | Weaver, R. G.', 'Hunt, E.', 'Armstrong, B.', 'Beets, M. W.', 'Brazendale, K.', 'Turner-McGrievy, G.', 'Pate, R. R.', 'Maydeu-Olivares, A.', 'Saelens, B.', 'Youngstedt, S. D.', 'Dugger, R.', 'Parker, H.', 'von Klinggraeff, L.', 'Jones, A.', 'Burkhart, S.', 'Ressor-Oyer, L.' | 2021 | Wrong intervention |
| Impact of Citizenship Education on the Civic Consciousness of Nigerian Youth | Iyamu, Ede O. S.', 'Obiunu, Jude J.' | 2005 | Wrong outcomes |
| The impact of free access to swimming pools on children's participation in swimming. A comparative regression discontinuity study | Higgerson, J.', 'Halliday, E.', 'Ortiz-Nunez, A.', 'Barr, B.' | 2019 | Wrong outcomes |
| The impact of summer vacation on childrenâ€™s obesogenic behaviors and body mass index: a natural experiment | Weaver, R. G.', 'Armstrong, B.', 'Hunt, E.', 'Beets, M. W.', 'Brazendale, K.', 'Dugger, R.', 'Turner-McGrievy, G.', 'Pate, R. R.', 'Maydeu-Olivares, A.', 'Saelens, B.', 'Youngstedt, S. D.' | 2020 | Wrong study design |
| Impact of Year-Round and Traditional School Schedules on Summer Weight Gain and Fitness Loss | Brusseau, T. A.', 'Burns, R. D.', 'Fu, Y.', 'Glenn Weaver, R.' | 2019 | Wrong intervention |
| Implications of race and ethnicity for child physical activity and social connections at summer care programs | Prochnow, T.', 'Patterson, M. S.', 'Hartnell, L.', 'West, G.', 'Umstattd Meyer, M. R.' | 2021 | Wrong study design |
| Improving social skills in latency-age children with emotional disturbances through increased ethnic identity | Huey, Shontinese Cooper' | 2006 | Wrong literature type |
| Increasing girls' physical activity during an organised youth sport basketball program: a randomised controlled trial protocol | Guagliano, Justin M.', 'Lonsdale, Chris', 'Kolt, Gregory S.', 'Rosenkranz, Richard R.' | 2014 | Wrong literature type |
| Individual, social, physical environmental, and organizational correlates of children's summer camp-based physical activity | Hickerson, Benjamin D.' | 2010 | Wrong literature type |
| Influence of school holidays on weight of children participating in a tertiary hospital weight management programme | Rao, S.', 'Alexander, S.' | 2011 | Wrong literature type |
| Injury and Illness Benchmarking and Prevention for Children and Staff Attending U.S. Camps: Promising Practices and Policy Implications | Garst, Barry A.', 'Erceg, Linda E.', 'Walton, Edward' | 2013 | Wrong population |
| An integrated components preventive intervention for aggressive elementary school children: The early risers program | August, G. J.', 'Realmuto, G. M.', 'Hektner, J. M.', 'Bloomquist, M. L.' | 2001 | Wrong population |
| Is a summer school programme a promising intervention in preparation for transition from primary to secondary school? | Siddiqui, N.', 'Gorard, S.', 'See, B. H.' | 2014 | Wrong outcomes |
| Learning problem-solving through making games at the game design and learning summer program | Akcaoglu, Mete' | 2014 | Wrong outcomes |
| Longitudinal achievement effects of multiyear summer school: Evidence from the Teach Baltimore randomized field trial | Borman, Geoffrey D.', 'Dowling, N. Maritza' | 2006 | Wrong study design |
| Making the Most of School Vacation: A Field Experiment of Small Group Math Instruction | Schueler, Beth E.' | 2020 | Wrong outcomes |
| Mental health awareness and mindfulness skills in primary school-aged children from ethnic minority backgrounds: A pilot health promotion programme | Aslam, A.', 'Hakim, A.', 'Ahmad, Z.' | 2019 | Wrong literature type |
| Metabolic health and academic achievement in youth at risk for high school dropout in rural Mississippi: The role of classroom management | Holmes, M. E.', 'Kvasnicka, M. A.', 'Brocato, D. K.', 'Webb, H. E.' | 2018 | Wrong intervention |
| A multi-method analysis of body mass index, physical activity, and executive functions among urban minority girls | Ward, Amanda K.' | 2016 | Wrong study design |
| Now Weâ€™re All Family: Exploring Social and Emotional Development in a Summer Hip Hop Mixtape Camp | Travis, R., Jr.', 'Levy, I. P.', 'Morphew, A. C.' | 2022 | Wrong outcomes |
| Nutrition impacts in a randomized trial of summer food benefits to prevent childhood hunger in U.S. schoolchildren | Briefel, R. R.', 'Collins, A. M.', 'Wolf, A.', 'Gordon, A. R.', 'Cabili, C. L.', 'Klerman, J. A.' | 2018 | Wrong study design |
| Obesity and physical fitness of pre-adolescent children during the academic year and the summer period: Effects of organized physical activity | Christodoulos, Antonios D.', 'Flouris, Andreas D.', 'Tokmakidis, Savvas P.' | 2006 | Wrong study design |
| Opportunities for promoting youth physical activity: An examination of youth summer camps | Hickerson, Benjamin D.', 'Henderson, Karla A.' | 2014 | Wrong study design |
| Outside-of-school time obesity prevention and treatment interventions in African American youth | Barr-Anderson, D. J.', 'Singleton, C.', 'Cotwright, C. J.', 'Floyd, M. F.', 'Affuso, O.' | 2014 | Wrong literature type |
| Parceling component effects of a multifaceted prevention program for disruptive elementary school children | August, G. J.', 'Egan, E. A.', 'Realmuto, G. M.', 'Hektner, J. M.' | 2003 | Wrong outcomes |
| A partnership approach to tackling inequalities through a summer holiday enrichment programme | Holmes, E.', 'Palmer, K.' | 2016 | Wrong literature type |
| Patterns and Temporal Changes in Peer Affiliation among Aggressive and Nonaggressive Children Participating in a Summer School Program | Hektner, J. M.', 'August, G. J.', 'Realmuto, G. M.' | 2000 | Wrong study design |
| Physical activity in middle school-aged children participating in a school-based recreation program | Kien, C. L.', 'Chiodo, A. R.' | 2003 | Wrong study design |
| A pilot study evaluating the feasibility of a summer gardening program to prevent summer weight gain in overweight adolescents | Jacquart, S. R.', 'Schoeller, D. A.', 'Adams, A. K.', 'Larson, N.', 'Dennis, S. F.', 'LaRowe, T. L.', 'Carrel, A. L.' | 2010 | Wrong literature type |
| Pilot study: Effects of short-term summer school program on plasma cognitive marker and non-lipid cardiovascular risk factors in female adolescents | Choi, M. D.', 'Park, K. S.' | 2017 | Wrong literature type |
| Pilot testing of an intensive cooking course for New Zealand adolescents: The create-our-own kai study | Black, K.', 'Thomson, C.', 'Chryssidis, T.', 'Finigan, R.', 'Hann, C.', 'Jackson, R.', 'Robinson, C.', 'Toldi, O.', 'Skidmore, P.' | 2018 | Wrong outcomes |
| Preparation For Medical School via an Intensive Summer Program for Future Doctors: A Pilot Study of Student Confidence and Reasoning Skills | Musick, David W.', 'Ray, Richard H.' | 2016 | Wrong population |
| Process evaluation of an up-scaled community based child obesity treatment program: NSW Go4Fun | Welsby, D.', 'Nguyen, B.', O'Hara, B. J., 'Innes-Hughes, C.', 'Bauman, A.', 'Hardy, L. L.' | 2014 | Wrong intervention |
| Process evaluation of an up-scaled community based child obesity treatment program: NSW Go4Fun R | Welsby, Debra', 'Nguyen, Binh', O'Hara, Blythe J., 'Innes-Hughes, Christine', 'Bauman, Adrian', 'Hardy, Louise L.' | 2014 | Wrong intervention |
| A Professional Development School--Sponsored Summer Program for At-Risk Secondary Students | Cuddapah, Jennifer L.', 'Masci, Frank J.', 'Smallwood, Jo Ellen', 'Holland, Jennifer' | 2008 | Wrong outcomes |
| Program development of a community based therapeutic day camp for children classified as emotionally disturbed | Bailey, Melisa B.' | 2010 | Wrong literature type |
| The programme 'Smoking and Me' has exerted its impact on elementary schoolchildren already for three years | Hruba, D.', 'Kachlik, P.' | 2000 | Wrong study design |
| Promoting physical activity through walking to treat childhood obesity, mainly for mild to moderate obesity | Yoshinaga, M.', 'Miyazaki, A.', 'Aoki, M.', 'Ogata, H.', 'Ito, Y.', 'Hamajima, T.', 'Tokuda, M.', 'Lin, L.', 'Horigome, H.', 'Takahashi, H.', 'Nagashima, M.' | 2020 | Wrong intervention |
| Promotion of physical activity and adequate nutrition in children during the summer school holidays | Perez-Lizaur, A. B.', 'Melendez-Mier, G.', 'Rocha-Aguilar, R.', 'Haua-Navarro, K.', 'Perez-Rodriguez, M.', 'Pffefer, F.' | 2011 | Wrong literature type |
| A rationale for the use of anthropometric measurements and bioelectrical impedance analysis as efficacy criteria for summer camp healthcare | Gavryushin, M. Yu', 'Sazonova, O. V.', 'Gorbachev, D. O.', 'Borodina, L. M.', 'Frolova, O. V.', 'Tupikova, D. S.' | 2019 | Wrong study design |
| Rebound Body Mass Index Growth in Year-Round Elementary Education Students of Largely Hispanic Descent Undergoing Obesity Interventions | Alexander, A. G.', 'Lyons, P. E.' | 2016 | Wrong intervention |
| S.P.L.A.S.H. into fitness! an identity-focused behavioral swim camp and family-oriented ehealth intervention for girls | Kramer, Eydie Noelle' | 2020 | Wrong literature type |
| School Supplies and Financial Literacy for Families in Poverty | Morris, Ronald V.', 'Shockley, Denise' | 2022 | Wrong literature type |
| School-based fitness changes are lost during the summer vacation | Carrel, Aaron L.', 'Clark, R. Randall', 'Peterson, Susan', 'Eickhoff, Jens', 'Allen, David B.' | 2007 | Wrong intervention |
| School-based mental health prevention activities for homeless and at-risk youth | Nabors, L.', 'Proescher, E.', 'DeSilva, M.' | 2001 | Wrong outcomes |
| School-Based Weight Management Program Curbs Summer Weight Gain Among Low-Income Hispanic Middle School Students | Reesor, L.', 'Moreno, J. P.', 'Johnston, C. A.', 'Hernandez, D. C.' | 2019 | Wrong intervention |
| School's out: What are urban children doing? the summer activity study of somerville youth (SASSY) | Tovar, A.', 'Lividini, K.', 'Economos, C. D.', 'Folta, S.', 'Goldberg, J.', 'Must, A.' | 2010 | Wrong study design |
| Seasonal differences in patient retention and BNI changes in pediatric weight management treatment | Pike, G.', 'Boyer, K.', 'LeQuia, L.', 'Stratbucker, W.', 'Cadieux, A.', 'Silver, L.', 'Tucker, J.' | 2021 | Wrong literature type |
| Seasonal variability in body mass index change among children enrolled in the Pediatric Obesity Weight Evaluation Registry: A step in the right direction | Lane, T. S.', 'Sonderegger, D. L.', 'Binns, H. J.', 'Kirk, S.', 'Christison, A. L.', 'Novick, M.', 'Tucker, J.', 'King, E.', 'Wallace, S.', 'Brazendale, K.', 'Kharofa, R. Y.', 'Walka, S.', 'Heer, H. D. D.' | 2023 | Wrong intervention |
| Self-monitoring during whole group reading instruction: Effects among students with emotional and behavioral disabilities during summer school intervention sessions | Rafferty, L. A.' | 2012 | Wrong outcomes |
| Staking out the Successful Student | Brown, Christopher' | 2005 | Wrong study design |
| STEMming the Swell of Absenteeism in the Middle Years: Impacts of an Urban District Summer Robotics Program | Mac Iver, Martha Abele', 'Mac Iver, Douglas J.' | 2019 | Wrong outcomes |
| A summer nutrition benefit pilot program and low-income children's food security | Collins, Ann M.', 'Klerman, Jacob A.', 'Briefel, Ronette', 'Rowe, Gretchen', 'Gordon, Anne R.', 'Logan, Christopher W.', 'Wolf, Anne', 'Bell, Stephen H.' | 2018 | Wrong outcomes |
| Summer School Effects in a Randomized Field Trial | Zvoch, Keith', 'Stevens, Joseph J.' | 2013 | Wrong population |
| Supporting at-risk youth and their families to manage and prevent diabetes: Developing a national partnership of medical residency programs and high schools | Gefter, L.', 'Morioka-Douglas, N.', 'Srivastava, A.', 'Rodriguez, E.' | 2016 | Wrong study design |
| Thinking outside the meals: How a community collaborative summer meals program influenced nutrition knowledge and skills, physical activity, and social interaction among Mexican-origin children | Sharkey, J.', 'Valdez, E.', 'Beltran, E.', 'Beltran, D.', 'Bustillos, B.' | 2014 | Wrong literature type |
| Transformative Performing Arts and Mentorship Pedagogy: Nurturing Developmental Relationships in a Multidisciplinary Dance Theatre Program for Youth | Kane, Kevin M.' | 2014 | Wrong study design |
| Treating childhood obesity by walking: A randomised controlled trial | Yoshinaga, M.', 'Seki, S.', 'Ogata, H.', 'Ito, Y.', 'Aoki, M.', 'Miyazaki, A.', 'Tokuda, M.', 'Lin, L.', 'Horigome, H.', 'Nagashima, M.' | 2017 | Wrong intervention |
| Understanding physical activity patterns among rural Aboriginal and non-Aboriginal young people | Macniven, R.', 'Richards, J.', 'Turner, N.', 'Blunden, S.', 'Bauman, A.', 'Wiggers, J.', 'Gwynn, J.' | 2019 | Wrong study design |
| University-School Partnerships: On the Impact on Students of Summer Schools (for School Students Aged 17-18) Run by Bristol ChemLabs | Shaw, A. J.', 'Harrison, T. G.', 'Croker, S. J.', 'Medley, M.', 'Sellou, L.', 'Shallcross, K. L.', 'Williams, S. J.', 'Grayson, D. J.', 'Shallcross, D. E.' | 2010 | Wrong population |
| An urban garden initiative: A component of project healthy schools | Wei, W. C. R.', 'Heeres, A.', 'Aaronson, S.', 'Rogers, R.', 'Lee, A.', 'Pew, A.', 'Foti, A.', 'Vuong, B.', 'Corriveau, N.', 'Jiang, Q.', 'Kline-Rogers, E.', 'Goldberg, C.', 'DuRussel-Weston, J.', 'Jackson, E. A.', 'Eagle, K. A.' | 2014 | Wrong literature type |
| Use of the School Setting During the Summer Holidays: Mixed-Methods Evaluation of Food and Fun Clubs in Wales | Morgan, Kelly', 'McConnon, Linda', 'Van Godwin, Jordan', 'Hawkins, Jemma', 'Bond, Amy', 'Fletcher, Adam' | 2019 | Wrong study design |
| Video game intervention for sexual risk reduction in minority adolescents: randomized controlled trial | Fiellin, L. E.', 'Hieftje, K. D.', 'Pendergrass, T. M.', 'Kyriakides, T. C.', 'Duncan, L. R.', 'Dziura, J. D.', 'Sawyer, B. G.', 'Mayes, L.', 'Crusto, C. A.', 'Forsyth, B. W. C.', 'Fiellin, D. A.' | 2017 | Wrong intervention |
| Youth empowerment solutions for violence prevention | Reischl, T. M.', 'Zimmerman, M. A.', 'Morrel-Samuels, S.', 'Franzen, S. P.', 'Faulk, M.', 'Eisman, A. B.', 'Roberts, E.' | 2011 | Wrong literature type |
| A residential summer camp can reduce body fat and improve health-related quality of life in obese children | Wong, William W. Barlow, Sarah E. Mikhail, Carmen Wilson, Theresa A. Hernandez, Paula M. Shypailo, Roman J. Abrams, Stephanie H. | 2013 | Wrong study design |
| Increasing Physical Activity and Enjoyment Through Goal-Setting at Summer Camp | Wilson, Cait Sibthorp, Jim Brusseau, Timothy A. | 2017 | Wrong study design |
| First year physical activity findings from turn up the HEAT (Healthy Eating and Activity Time) in summer day camps | Weaver, R. Glenn Brazendale, Keith Chandler, Jessica Turner-McGrievy, Gabrielle Moore, Justin B. Huberty, Jennifer Ward, Dianne S. Beets, Michael W. | 2017 | Wrong study design |
| Comparing Campers' Physical Activity Levels Between Sport Education And Traditional Instruction in a Residential Summer Camp | Wahl-Alexander, Zachary Morehead, Craig A. | 2017 | Wrong study design |
| Ya gotta have friends: Social support and self-efficacy predict success following immersion treatment | Sampat, Sonia Kirschenbaum, Daniel S. Gierut, Kristen J. Germann, Julie N. Krawczyk, Ross | 2014 | Wrong study design |
| Camp Jump Start: Effects of a Residential Summer Weight-Loss Camp for Older Children and Adolescents | Huelsing, Jean Kanafani, Nadim Mao, Jingnan White, Neil H. | 2010 | Wrong study design |
| Camp NERF: Feasibility, Acceptability, and Potential Efficacy of a Theory-Based Nutrition Education Recreation and Fitness Program Aimed at Preventing Unhealthy Weight Gain in Disadvantaged Children during Summer Months | Hopkins, Laura Rose, Angela Gunther, Carolyn | 2015 | Wrong literature type |
| Camp NERF: Efficacy of a Theory-Based Nutrition Education Recreation and Fitness Program Aimed at Preventing Unhealthy Weight Gain in Disadvantaged Children during Summer Months | Hopkins, Laura Gunther, Carolyn | 2016 | Wrong literature type |
| Opportunities for Promoting Youth Physical Activity: An Examination of Youth Summer Camps | Hickerson, Benjamin Henderson, Karla A. | 2013 | Wrong study design |
| Influence of Session Context on Physical Activity Levels Among Russian Girls During a Summer Camp | Guagliano, Justin M. Updyke, Natalie J. Rodicheva, Natalia V. Rosenkranz, Sara K. Dzewaltowski, David A. Schlechter, Chelsey R. Rosenkranz, Richard R. | 2017 | Wrong study design |
| The effects of a children's summer camp programme on weight loss, with a 10 month follow-up | Gately, Paul Cooke, Carlton Butterly, R. J. Mackreth, P. Carroll, Sean | 2000 | Wrong study design |
| For comparison: experience with a childrenâ€™s obesity camp | Cooper, Christopher Sarvey, Sharon I. Collier, David N. Willson, Charles F. Green, Ira Pories, Mary Lisa Rose, Mary Ann Escott-Stump, Sylvia Pories, Walter J. | 2006 | Wrong study design |
| Camp-Based Immersion Treatment for Obese, Low Socioeconomic Status, Multi-Ethnic Adolescents | Carraway, Marissa Lutes, Lesley D. Crawford, Yancey Pratt, Keeley J. McMillan, Amy Gross Scripture, Lee G. Henes, Sarah T. Cox, James Vos, Paul Collier, David N. | 2014 | Wrong study design |
| Maximizing children's physical activity using the LET US Play principles | Brazendale, Keith Chandler, Jessica Beets, Michael W. Weaver, Robert G. Beighle, Aaron Huberty, Jennifer Moore, Justin B. | 2015 | Wrong study design |
| Childrenâ€™s Moderate to Vigorous Physical Activity Attending Summer Day Camps | Brazendale, Keith Beets, Michael W. Weaver, R. Glenn Chandler, Jessica Randel, Allison B. Turner-McGrievy, Gabrielle Moore, Justin B. Huberty, Jennifer Ward, Dianne S. | 2017 | Wrong study design |
| Preventing Summer Learning Loss: Results of a Summer Literacy Program for Students from Low-SES Homes | Bowers, Lisa M. Schwarz, Ilsa | 2017 | Wrong outcomes |
| Improving Urban Minority Girlsâ€™ Health Via Community Summer Programming | Bohnert, Amy M. Bates, Carolyn R. Heard, Amy Burdette, Kimberly A. Ward, Amanda K. Silton, Rebecca L. Dugas, Lara R. | 2017 | Wrong study design |
| How physically active are children attending summer day camps | Beets, Michael W. Weaver, Robert G. Beighle, Aaron Webster, Collin A. Pate, Russell R. | 2012 | Wrong study design |
| The Fun, Food, and Fitness Project (FFFP): the Baylor GEMS pilot study | Baranowski, Tom Baranowski, Janice Cullen, Karen W. Thompson, Deborah Nicklas, Theresa A. Zakeri, Issa Rochon, James | 2003 | Wrong study design |
