# Supplementary material for: Health effects of children’s summer holiday programs: a systematic review and meta-analysis
Source: Int J Behav Nutr Phys Act. 2024 Oct 18;21:119. doi: 10.1186/s12966-024-01658-8 (PMC11488216; doi:10.1186/s12966-024-01658-8)
Supplement: Supplementary file 7 — Supplementary Material 7: Supplementary File 6: Thematic Coding of Program Characteristics [file 12966_2024_1658_MOESM7_ESM.docx]

**Supplementary File 6: Thematic Coding of Program Characteristics**

| **Study** | **Participant characteristics** | | **Program characteristics** | | | | | | | |  |
| --- | --- | --- | --- | --- | --- | --- | --- | --- | --- | --- | --- |
|  | **Age** | **SES** | **Format** | **Setting** | **Content** | **Cost** | **Dura-tion (w)** | **Daily contact** | **Inten-sity** | **Enrichment** | **Attend-ance** |
| **D'Haese 2015** | Primary | Mixed | Day | Community | General | Free | 1 | Half day | Daily | Local council provided activities and games | High |
| **Dugger 2020** | Primary | Low | Day | School | Specific: Academic and PA and diet | Funded | 6 | Full day | Daily | M | High |
| **Evans 2018** | Primary | Low | Day | Community | Specific: PA and diet | Funded | 8 | Half day | Daily | Extra daily activities: karate, lego, obstacles, hip-hop, creative movement. M. | High |
| **Evans 2020** | Primary | Low | Day | Community | Specific: PA and diet | Funded | 7 | Full day | Daily | Games and activities incorporated into the program. M, T. | Moderate |
| **Gately 2005** | Mixed | Mixed | Residential | School | Specific: PA and diet | Paid | 2 to 6 | Full day | Not reported | M | NR |
| **Hazar 2019** | Primary | NR | Day | School | Specific: PA and diet | Not reported | 8 | Sessional | Daily | M | NR |
| **Matsui 2019** | Primary | NR | Day | School & Home | Specific: PA | Not reported | 6 | Sessional | Occasional |  | NR |
| **Meucci 2013** | Primary | NR | Day | NR | Specific: PA and diet | Funded | 4 to 8 | School day | Not reported | M | NR |
| **Park 2015** | High | Low | Day | School | Specific: PA and diet | Not reported | 5 | Full day | Daily | M | NR |
| **Von Klinggraeff 2022** | Primary | Low | Day | School | Specific: PA and diet | Funded | 6 | Full day | Daily | M, T | Moderate |

Legend: M: reported that meals were provided. NR: Not reported. T: reported that transport was provided. W: weeks,

**Explanation of Thematic Coding**

| **Item** | **Explanation** | **Code** |
| --- | --- | --- |
| *Participant characteristics* | | |
| Age | 1. Based on mean age of participants: Year/Grade levels:  - R/K-2 (5-7 yrs): Lower primary - G3-5 (8-10 yrs): Mid primary - G6-8 (11-13 yrs): Upper primary - G9+ (14+ yrs): High school  1. Mixed: More than one age group 2. If mean not reported, based on the age range of 75% of participants 3. If more than one category = mixed | - Lower primary - Mid primary - Upper primary - High school - Mixed |
| SES | SES of the population   - Low: Low SES, “underprivileged” - High: High SES, “privileged” - Mixed: more than one SES band | - Low - High - Mixed |
| *Program characteristics: Structure* | | |
| Type | Residential, day or home program:   - Residential includes accommodation with overnight stays - Day programs are conducted outside of the home with children returning home at the end of every day - Home programs are conducted at home, with/without an initial introductory/measurement session. - Mixed programs are conducted in more than one setting | - Residential - Day - Home - Mixed |
| Setting | Place the program is delivered:   - Community: including public spaces, community organisations - School: educational facilities including schools, boarding schools, summer schools or universities - Home | - Community - School - Home |
| Content | Specific program or general   - Specific (curriculum designed to address specific skill or behaviour) - General (enrichment – program centred around games, toys, play or recreation) | - Specific - General |
| Cost | Cost to participants (program funding):   - Free: no charge to participants e.g., program already established and externally funded - Funded: no charge to participants as the program was funded for a brief period  e.g., trial funding - Paid: Participants paid to attend. Full or partial scholarships may have been available to some participants. | - Free - Funded - Paid |
| *Program characteristic: Contact* | | |
| Duration | Time between first and last program session. Where programs had options for attendance (e.g., four weeks or eight weeks, these are stated as a range) | Number of weeks |
| Daily contact time | Contact hours per day:   - Sessional (2 hours or less per visit) - Half day (>2 & <6 hours) - School day (6-7 hours) - Full day (>7 hours) day | - Sessional - Half day - School - Full day |
| Intensity | Average Number of face-to-face contact sessions with the program per week. Calculated by dividing the number of sessions by the total weeks (start to finish). Occasional programs may have had one workshop session per month or two formal sessions throughout summer combined with homework. | - Daily (4-5 x week) - Bi-weekly (2x week), - Occasional (1 or less per week) |
| Other features | Factors that were reported and that supported attendance. Extra enrichment activities listed. Specifically, if meals (M) and transport to and from the program (T) were reported. | - M: Meals - T: Transport |
| Attendance | Attendance level recorded and reported as % of scheduled sessions.   - 50% (low) - 51-65% (moderate) - 66-100% (high) | - Low - Moderate - High |

Key: G: Grade. SES: Socioeconomic Status
