# Supplementary material for: Health effects of children’s summer holiday programs: a systematic review and meta-analysis
Source: Int J Behav Nutr Phys Act. 2024 Oct 18;21:119. doi: 10.1186/s12966-024-01658-8 (PMC11488216; doi:10.1186/s12966-024-01658-8)
Supplement: Supplementary file 8 — Supplementary Material 8: Supplementary File 7: Quality appraisal of included studies [file 12966_2024_1658_MOESM8_ESM.docx]

**Supplementary File 7: Quality appraisal of included studies**

**PEDro Results**

| **First Author (year)** | **Item 1*** | **Item 2** | **Item 3** | **Item 4** | **Item 5** | **Item 6** | **Item 7** | **Item 8** | **Item 9** | **Item 10** | **Item 11** |
| --- | --- | --- | --- | --- | --- | --- | --- | --- | --- | --- | --- |
| D'Haese (2015) | yes | no | no | no | no | no | no | no | yes | yes | yes |
| Dugger (2020) | yes | yes | yes | no | no | no | no | yes | yes | yes | yes |
| Evans (2018) | yes | no | no | no | no | no | no | no | yes | yes | yes |
| Evans (2020) | yes | yes | yes | yes | no | no | yes | yes | yes | yes | yes |
| Gately (2005) | yes | no | no | no | no | no | no | no | no | yes | yes |
| Hazar (2019) | no | no | no | yes | no | no | no | yes | no | yes | no |
| Matsui (2019) | no | no | no | yes | no | no | no | yes | yes | yes | yes |
| Meucci (2013) | yes | yes | no | no | no | no | no | yes | yes | yes | yes |
| Park (2015) | yes | no | no | yes | no | no | no | yes | no | yes | yes |
| vonKlinggraeff (2022) | yes | yes | no | yes | no | no | no | yes | yes | yes | yes |

| \| **First Author (year)** \| **PEDro Score** \| **Risk of Bias** \| \| --- \| --- \| --- \| \| D'Haese (2015) \| 3 \| High \| \| Dugger (2020) \| 6 \| Moderate \| \| Evans (2018) \| 3 \| High \| \| Evans (2020) \| 8 \| Low \| \| Gately (2005) \| 2 \| High \| \| Hazar (2019) \| 3 \| High \| \| Matsui (2019) \| 5 \| Moderate \| \| Meucci (2013) \| 5 \| Moderate \| \| Park (2015) \| 4 \| High \| \| vonKlinggraeff (2022) \| 6 \| Moderate \| | * Item 1 is not computed in the final score^1^  Maximum possible PEDro score: 10  Adjusted maximum PEDro score: 8  **PEDro categories:**  0-4: Low  5-6: Moderate  7-8: High |
| --- | --- | --- | --- | --- | --- | --- | --- | --- | --- | --- | --- | --- | --- | --- | --- | --- | --- | --- | --- | --- | --- | --- | --- | --- | --- | --- | --- | --- | --- | --- | --- | --- | --- | --- |

**PEDro Items**

| 1 | Inclusion/Exclusion: Eligibility criteria were specified |
| --- | --- |
| 2 | Random Allocation: Subjects were randomly allocated to groups (in a crossover study, subjects were randomly allocated an order in which treatments were received) |
| 3 | Allocation Concealment: Allocation was concealed |
| 4 | Baseline Similarities: The groups were similar at baseline regarding the most important prognostic indicators |
| 5 | Blinding of Participants: There was blinding of all subjects |
| 6 | Blinding of Therapist/Deliverer of Intervention: There was blinding of all therapists who administered the therapy |
| 7 | Blinding of Outcome Assessment: there was blinding of all assessors who measured at least one key outcome |
| 8 | Incomplete Data/Dropouts: Measures of at least one key outcome were obtained from more than 85% of the subjects initially allocated to groups. |
| 9 | Intention to Treat Analysis: All subjects for whom outcome measures were available received the treatment or control condition as allocated or, where this was not the case, data for at least one key outcome was analysed by intention to treat |
| 10 | Testing the Difference Between Groups is Greater than Chance: The results of between-group statistical comparisons are reported for at least one key outcome |
| 11 | Effect Size and Certainty: The study provides both point measures and measures of variability for at least one key outcome |

~Considering intervention type studied, the following items are difficult to achieve, therefore the maximum achievable PEDro score may be considered to be lower^2^. Such items include:

- Item 5: Blinding of Participants
- Item 6: Blinding of Therapist/Deliverer

1. Paci M, Bianchini C, Baccini M. Reliability of the PEDro scale: comparison between trials published in predatory and non-predatory journals. *Archives of Physiotherapy*. 2022;12(1):1-9.

2. Cashin AG, McAuley JH. Clinimetrics: Physiotherapy Evidence Database (PEDro) Scale. *J Physiother*. Jan 2020;66(1):59. doi:10.1016/j.jphys.2019.08.005
